# A simple metric of promoter architecture robustly predicts expression breadth of human genes suggesting that most transcription factors are positive regulators

**DOI:** 10.1186/s13059-014-0413-3

**Published:** 2014-07-31

**Authors:** Laurence D Hurst, Oxana Sachenkova, Carsten Daub, Alistair RR Forrest, the FANTOM consortium, Lukasz Huminiecki

**Affiliations:** Department of Biology and Biochemistry, University of Bath, Bath, BA2 7AY UK; Department of Biochemistry and Biophysics, Stockholm University, Stockholm, Sweden; Science for Life Laboratory, SciLifeLab, Stockholm, Sweden; RIKEN Omics Science Center, Yokohama, Japan; Department of Cell and Molecular Biology, Karolinska Institutet, Stockholm, Sweden; BILS bioinformatics infrastructure for life sciences, Stockholm, Sweden; Department of Immunology Genetics and Pathology, Uppsala University, Uppsala, Sweden; Division of Genomic Technologies, RIKEN Center for Life Science Technologies, Yokohama, Kanagawa Japan

## Abstract

**Background:**

Conventional wisdom holds that, owing to the dominance of features such as chromatin level control, the expression of a gene cannot be readily predicted from knowledge of promoter architecture. This is reflected, for example, in a weak or absent correlation between promoter divergence and expression divergence between paralogs. However, an inability to predict may reflect an inability to accurately measure or employment of the wrong parameters. Here we address this issue through integration of two exceptional resources: ENCODE data on transcription factor binding and the FANTOM5 high-resolution expression atlas.

**Results:**

Consistent with the notion that in eukaryotes most transcription factors are activating, the number of transcription factors binding a promoter is a strong predictor of expression breadth. In addition, evolutionarily young duplicates have fewer transcription factor binders and narrower expression. Nonetheless, we find several binders and cooperative sets that are disproportionately associated with broad expression, indicating that models more complex than simple correlations should hold more predictive power. Indeed, a machine learning approach improves fit to the data compared with a simple correlation. Machine learning could at best moderately predict tissue of expression of tissue specific genes.

**Conclusions:**

We find robust evidence that some expression parameters and paralog expression divergence are strongly predictable with knowledge of transcription factor binding repertoire. While some cooperative complexes can be identified, consistent with the notion that most eukaryotic transcription factors are activating, a simple predictor, the number of binding transcription factors found on a promoter, is a robust predictor of expression breadth.

**Electronic supplementary material:**

The online version of this article (doi:10.1186/s13059-014-0413-3) contains supplementary material, which is available to authorized users.

## Background

Is it possible to predict expression parameters of a gene from knowledge of the promoter architecture of that gene? If, for example, we knew the transcription factors (TF) that bind the promoter of a gene, can we predict the breadth of expression (BoE) (that is, the proportion of tissues/cells within which the gene is expressed) or the mean level of expression of that gene? It is known that expression patterns of gene duplicates diverge over evolutionary time [1,2], but can we predict how different the expression of paralogs will be knowing nothing more than their promoter architecture? What in turn is the relationship between expression breadth and the number of TFs regulating a gene (TfbsNo.)? Given that, in contrast to prokaryotes, the ground state for most eukaryotic genes is inactivity [3], we might expect that broadly expressed genes should have very many regulating TFs, assuming eukaryotic TFs are for the most part activating [4]. However, some very broadly expressed genes might have reverted to a more prokaryotic state and have activity as the constitutive state and hence not require TF activation. Alternatively, the BoE may be conferred by the ability to bind a few specialist transcription factors or through cooperation of particular TFs, in which case the total number of binders need not predict breadth.

At first sight the answer to many of these questions may appear rather trivial: surely if we know the TFs that bind a gene’s promoter and know when those TFs are present in cells then we must know the expression parameters of a gene [5]? However, an in-depth study of STE12 found that expression changes in response to this transcription factor accounted for only half the observed expression fluctuations [6]. That the coupling between TF presence/absence need not be such an excellent predictor is indicative of other levels of control. In addition to transcription level regulation (presence/absence of the relevant TFs), genes can be regulated both pre- and post-transcriptionally. Post-transcriptionally, processes such as nonsense-mediated decay (NMD) [7], microRNA level regulation [8], and modulation of RNA stability [9], can also act to reduce the transcript levels below that expected given the transcription rate, potentially buffering larger changes in mRNA levels. Chromatin level pre-transcriptional regulation may be the dominant factor [10]. This can mean either higher-level chromatin architecture (open/closed chromatin configuration) [10] or other epigenetic marks (histone modification, methylation, and so on) [11,12], all of which can modulate the expression of the gene even if the relevant TFs are present.

Much evidence supports a strong role for chromatin in dictating expression profiles. For example, insertion of the same transgene into different regions in the genome leads to different expression levels dependent on the expression profile of the neighboring genes [13]. Similarly, a pair of transgenes can be co-expressed if introduced in tandem (so sharing the same chromatin environment) but have uncoordinated expression when introduced into unlinked locations [14]. Upregulation of one gene is similarly thought to cause a time-lagged ripple of chromatin opening which leads to spikes in the expression of neighbors [15]. More generally, at least in yeast, physical proximity of genes, is a strong predictor of the degree of co-expression between any two genes [16]. Indeed, for unlinked genes, on average two genes with the identical repertoire of TF binders, have only a weak degree of co-expression (*r*^*2*^ approximately 1% to 2%), much less than the degree of co-expression of two linked genes with no transcription factors in common (*r*^*2*^ approximately 10%) [16]. Moreover, DNA methylation was found to increase or decrease BoE depending on the target sequence [17]; while CpG islands co-localize with most promoters and are characterized by low methylation [18]. These results all suggest that chromatin level effects are not negligible and that extrapolation from TF binding to expression profile might be a relatively futile enterprise. In contrast to this position, however, is a striking counter-example demonstrating that the expression profile of genes involved in *Drosophila* segmentation is well predicted by the knowledge of TF binding sites and TF levels [5].

One approach to determine the extent to which promoter architecture determines expression parameters has been to consider the relationship between expression divergence and promoter divergence between paralogs within a genome or between orthologs in different genomes [19–22]. The logic is the same in both instances, namely that if the differences from the ancestral expression profile to current expression profile have been owing to changes in the sequence of the promoters, then comparing multiple genes across genomes (for orthologs) or within genomes (for paralogs) should reveal correlations between the degree of promoter divergence and the degree of expression divergence. In the instance of paralogs there is an additional assumption that the duplicate versions of the same gene were generated in a manner that preserved the promoters. These analyses commonly suggest little or no coupling between promoter divergence and expression divergence, consistent with a weak coupling between promoter architecture and gene expression parameters. For example, within yeasts divergence of transcription factor binding sites (Tfbs) has little impact on expression divergence between orthologs [19]. Similarly, Park and Makova found in humans that the correspondence of paralog *cis*-regulatory regions was so weakly correlated with expression divergence in a multiple regression that it was not significant after multi-test correction [20]. A further yeast study found that promoter divergence explained only 2% to 3% of expression variability [21]. These results suggest that *cis*-regulatory effects are not a major influence on expression profile. By contrast, a promoter screen in yeast found evidence for a robust correlation between the number of shared motifs and the degree of expression divergence between paralogs [22], although, unexpectedly, the absolute number of motifs the paralogs have is approximately constant over time. Clearly, more analysis is needed to investigate this key question in the field of expression pattern evolution.

While the consensus view is that promoter architecture does not well predict expression parameters, there is also then a lack of perfect agreement on this. One possible reason the studies are not obviously in agreement is that there is much noise in both measures of expression and inference of which proteins bind any given gene’s promoter. In addition, it is not immediately clear what metric of, for example, promoter divergence would be most informative. We return to this issue employing a merge of two exceptional data sources, ENCODE and FANTOM5. We used ENCODE ChIP-seq meta dataset derived from multi cell-line clustered experiments published in 2012 [23]. Whole-genome studies of regulatory evolution in human had been unfeasible before ENCODE [23]. Although ENCODE experiments were performed on separate cell lines, standardized experimental protocols and a unified analytical pipeline [24] allow one to merge ENCODE data into one meta dataset [25,26]. FANTOM5 is the most comprehensive expression dataset available, including 952 human and 396 mouse tissues, primary cells, and cancer cell lines (see Table 1). FANTOM5 [27] is based on cap analysis of gene expression (CAGE). CAGE characterizes transcriptional start sites across the entire genome in an unbiased fashion, and at a single-base resolution level [27].

**Table 1:** **The numbers of samples in distinct FANTOM5 categories**

	**Human**	**Mouse**
The total	952	396
Tissues	179	280
Primary cells	513	116
Cancer cell lines^a^	260	-
Brain tissues^b^	60	51
Reproductive tissues^c^	14	21

The first release of FANTOM5 included 952 human and 396 mouse tissues, primary cells and cancer cell lines. FANTOM5 explored the entire genome space in an unbiased and systematic fashion, without arbitrarily pre-selected features of the microarray chip. All FANTOM5 libraries passed strict quality control tests.

^a^Cancer cell lines are only available for human.

^b,c^Brain tissues and reproductive tissues are subsets of the tissue set.

Here, then, we employ this novel data to ask whether expression profiles can be predicted from promoter architecture. In the first instance we wish to know whether the total number of transcription factors binding a promoter is a good predictor. We follow this up with the analysis of interactants and a more complex machine learning approach. We start by resolving basic parameters of TF binding and promoter architecture.

## Results

### The number of transcription factors per gene follows a power law

**Figure 1:**
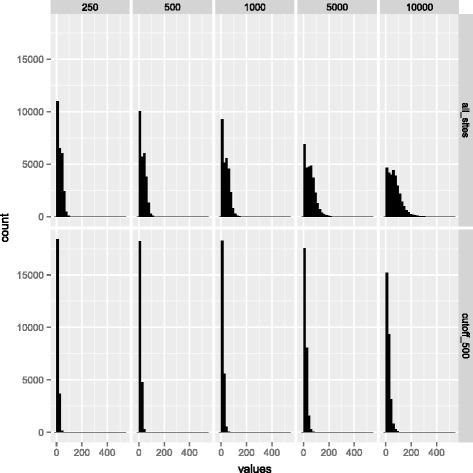
**Histograms of the numbers of Tfbs in promoter regions depending on analysis widow size and ENCODE quality cutoff.** This figure consists of 10 panels identified through row and column margin labels. The top row provides information on Tfbs distributions including all ENCODE sites‚ while the bottom row illustrates distributions at the ENCODE quality cutoff of 500. The motif quality cutoff refers to the quality score assigned to all Tfb sites by the ENCODE consortium, which are in the range of zero to 1,000 (from low to high quality). The promoter window sizes are in the range of 250 to 10,000 ± TSS (see column labels). The inclusion of all sites and the expansion of the analysis window result in distributions with longer tails in high numbers of mapping Tfbs.

Before attempting to describe any correlations between the number of Tfbs (TfbsNo.) and expression, it is instructive to know what the distribution of the number of transcription factors per gene looks like. Perhaps it is normally distributed? To determine this, proximal promoters were defined by a symmetrical window around the transcription start site – TSS (±500 bps). The distribution of TfbsNo. is not normal, instead it follows a power law (Figure 1). At the Tfbs quality cutoff of 500, 90% of genes had between 0 and 26 transcription factor binding sites, but there was a long-tail of genes with high values (more than 26). The distribution can be defined by Tukey’s five numbers: the minimum 0, the lower-hinge 0, the median 4, the upper hinge 14, and the maximum 58. The ENCODE motif quality cutoff refers to the quality score assigned to all Tfb sites and varying from zero through 1,000 [24], proportionately to the reliability of the predicted Tfbs. Additional details of the distribution of the number of Tfbs mapping to promoters with varied ENCODE quality cutoff and varied promoter window size are given in Tables 2 and 3.

**Table 2:** **The distribution parameters for the number of transcription factor binding sites mapping to proximal promoters depending on the promoter window size and ENCODE quality cutoff**

	**Size (bps)**	**ENCODE cutoff**	**Min**	**1st Qu.**	**Median**	**Mean**	**3rd Qu.**	**Max.**	**SD**
1	250	all_sites	1	8	23	25.92	41	109	19.69
2	250	cutoff_500	1	3	8	9.952	15	56	7.84
3	500	all_sites	1	9	28	30.6	48	126	23
4	500	cutoff_500	1	4	9	10.96	16	58	8.64
5	1,000	all_sites	1	11	33	35.93	55	162	27.17
6	1,000	cutoff_500	1	4	10	12	18	71	9.52
7	5,000	all_sites	1	20	47	51.75	74	368	38.96
8	5,000	cutoff_500	1	6	13	15.55	22	105	12.41
9	10,000	all_sites	1	29	61	68.75	95	515	51.34
10	10,000	cutoff_500	1	8	17	19.81	28	154	15.88

**Table 3:** **The percentages of genes with 1 TF, 2 TFs, and up to 5 TFs depending on the promoter window size and ENCODE quality cutoff**

	**Size (bps)**	**ENCODE cutoff**	**1 TF**	**2 TFs**	**Up to 5 TFs**
1	250	all_sites	6.04	4.49	19.84
2	250	cutoff_500	12.06	7.88	37.53
3	500	all_sites	5.44	3.85	17.54
4	500	cutoff_500	10.57	7.93	34.58
5	1,000	all_sites	4.6	3.17	15.18
6	1,000	cutoff_500	9.09	7.01	31.87
7	5,000	all_sites	1.88	1.56	8.33
8	5,000	cutoff_500	6.08	4.92	23.79
9	10,000	all_sites	0.9	0.76	4.45
10	10,000	cutoff_500	4.22	3.38	17.82

Values in the last three columns refer to a rate in each hundred.

### Effective promoter size is about 6 kb (±3,000 bps from the TSS)

**Figure 2:**
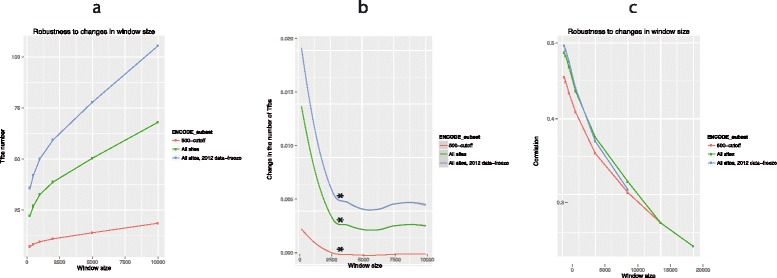
**Robustness to the variation in the size of the analysis window.** This figure consists of three parts identified as **(a - c)**. In **(a)**, the number of transcription factor binding sites depending on the size of the promoter window was shown. As expected, the number of Tfbs was increasing progressively with the window size. However, the rate of the increase gradually decreased and transformed to linear. The point of the transformation was the presumed boundary of the proximal promoter. To localize this boundary more precisely, we fitted a local polynomial regression (loess) model and plotted its first derivative in **(b)**. For all three subsets of ENCODE, there was a clear point of transformation where the rate of change (∆Tfbs) became constant (marked with ‘*’), at the distance from the TSS of approximately 3,000 base pairs (that is, the promoter window of 6,000 base pairs). Thus the outer boundary of promoters was estimated at 3,000 base pairs from the transcription start site (TSS). In **(c)**, we show that the correlation between the BoE and the number of transcription factor biding sites was robust to variation in window size, although its strength was decreasing as the size of the analysis window was increasing. This observation suggested that Tfbs controlling the BoE were enriched close to the transcription start site. Note that the analyses described here used either a 2011 or 2012 ENCODE data-freeze. The 2011 meta dataset included 2.7 million peaks for 148 transcription factors, derived from 71 cell types with 24 additional experimental cell culture conditions [31]. Peak scores varied from zero through 1,000. We used either all data or only high-quality peaks with the score above 500. The 2012 data-freeze, a broader dataset, consisted of 161 transcription factors and 91 human cell types with various treatment conditions [32].

We have assumed above a given size for promoters. Can we use our data to determine an average upper limit to the size of promoters? We expect that TF binding sites should be concentrated near the TSS and as we move ever further away the increase in the number of TF binding sites should tend to a linear function, indicating background/random rates. As expected, the number of Tfbs increases progressively with the window size, transforming gradually to a linear, background, rate of increase (Figure 2a). Using a derivative to determine the point at which the trend linearizes, the outer boundary of promoters is estimated at 3 kb from the TSS (Figure 2b).

### Broadly expressed genes have more transcription factor binding sites

**Figure 3:**
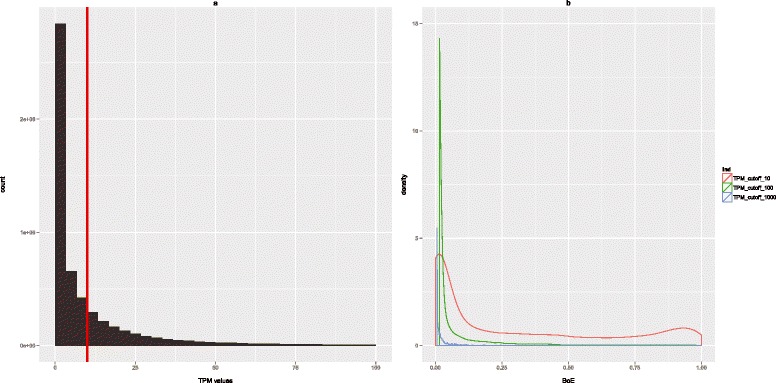
**The definition of the BoE.** This figure consists of two panels. **(a)** A histogram of all TPM values for human tissues. The following are the characteristics of the distribution: n = 5.566005e + 06, mean = 1.807312e + 01, median = 3.090820e + 00, sd = 317,803, min = 0, max = 348,120. The cutoff of 10 TPM is signified with the red vertical line. The BoE was the fraction of samples in which the gene was ‘on’, that is, in which it was transcribed. The tags per-million (TPM) value of 10 was accepted by the FANTOM5 consortium as the standard threshold for a gene to be ‘on’ in a given library. We considered alternative cutoffs of TPM = 100 and TPM = 1,000 in **(b)** which compares the density plots for the BoE at the cutoffs of 10, 100, and 1,000 TPM (see figure legend). It is clear that cutoffs of 100 and 1,000 are too high, resulting in almost no intermediate and housekeeping genes (that is, genes with BoE >0.33).

Is there something special about those genes with very many TF binding sites? Are they for example broadly expressed, as expected if TFs are dominantly activating? To analyze this we presumed, in the first instance, that a CAGE signal greater than 10 tags per million (TPM >10) classified a gene as expressed, or ‘on’ in a given tissue (this was the consensus definition accepted by the FANTOM5 consortium). The BoE is the fraction of tissues or cell-lines in which the gene was ‘on’, that is, in which it was transcribed. Figure 3 illustrates the distribution of TPM values in human tissues (Figure 3a), and the consequences of using too high a cutoff for BoE such as 100 or 1,000 TPM (Figure 3b). The TPM value of 10 is equivalent to approximately 3 mRNA copies per cell, based on 300,000 mRNAs per cell [28]. Using this definition, half of genes are relatively narrowly expressed. If, for example, transcripts are sub-divided into three categories, narrowly expressed (0 < the BoE ≤ 0.33), intermediate (0.33 < the BoE ≤ 0.66), and house-keeping (BoE >0.66), nearly half are tissue specific or narrowly expressed (0.46 narrowly expressed, 0.14 intermediate, and 0.21 housekeeping). Of the narrowly expressed transcripts, a very small fraction, 0.042 at the cutoff of 10 TPM or 0.053 at the cutoff of 100 TPM, are tissue-specific *sensu stricto*, that is, expressed in one tissue only. The remaining 0.19 is the fraction of transcripts which lack evidence for expression in FANTOM5 tissue samples at the cutoff of 10 TPM, owing perhaps to their highly restricted spatial and/or temporal expression in a very limited subset of cells. In comparison to all genes, ENCODE Tfbs have higher average BoE (BoE of 0.46 *versus* 0.295, Wilcoxon rank sum test *P* value = 2.995e-08) with the fractions of tissue-specific, intermediate, and housekeeping Tfbs at 0.32, 0.17, and 0.38. Top 10 housekeeping Tfbs included Pol2, JunD, c-Fos, JunB, Rad21, GTF2F1, NELFe, SREBP2, RXRA, and HSF1 (which all had BoE >0.98). For 17 Tfbs (that is, 12% of the total) we found no evidence of expression in tissue samples.

**Figure 4:**
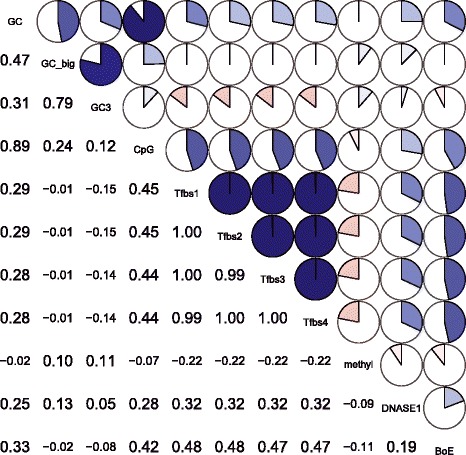
**A correlogram of 11 variables describing promoter architecture.** In the correlogram, there are four measures of GC-content: GC-content in a 1 kb proximal promoter (GC), GC-content in a 20 kbps window around the promoter (GC_big), GC-content in a third codon position (GC3), and the frequency of CpG sites (CpG); there are also four measures describing the number of transcription factor binding sites in promoters: Tfbs1 (*Tfbs_length --* straight number of Tfbs), Tfbs2 (*Tfbs_length_unique --* the number of unique Tfbs), Tfbs3 (*Tfbs_length_noPol2 --* the number of Tfbs excluding PolII), and Tfbs4 (*Tfbs_length_unique_noPol2 -* the number of unique Tfbs excluding PolII), a measure of methylation (methyl), a signature of digestion by DNASE1 (DNASE1), and the BoE.

**Figure 5:**
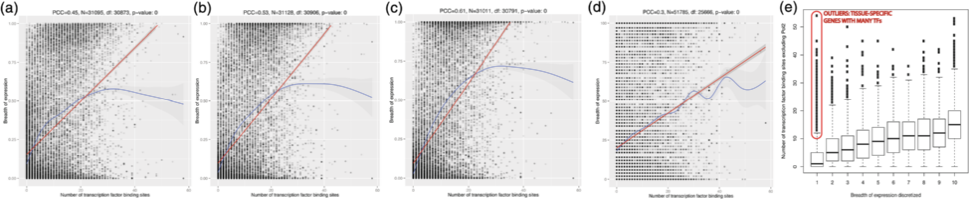
**The BoE correlated with the number of transcription factor binding sites.** The BoE correlated with the number of transcription factor binding sites in proximal promoters. Scatterplots were shown for **(a)** FANTOM5 human tissues, **(b)** FANTOM5 human primary cells, **(c)** FANTOM5 human cancer cell lines, **(d)** human data in Gene Expression Atlas [49]. The red line signified the linear model for the smoother line, while the blue line signified the non-linear model. **(e)** An alternative illustration of the trend using a boxplot for the discretized BoE in FANTOM5 tissues. Outlying tissue-specific genes with many transcription factor binding sites, which were likely enriched in inhibitory TFs, were marked in red. FANTOM5 tissues, primary cells, and cancer cell lines were the three subsets of samples in FANTOM5 whose numbers were given in Table 1. Numbers of tags in FANTOM5 were normalized to tags per million (TPM). The TPM value of 10 was chosen as a standard cutoff for a gene to be ‘on’. For Gene Expression Atlas, Affymetrix average difference (AD) higher that 200 classified a gene as ‘on’ or expressed in a given tissue. Proximal promoters were defined by a symmetrical window of 1 kb in size around the transcription start site (±500 bps from the TSS). As an additional control, we performed a randomization procedure where proximal promoters of all genes were shuffled. The value of the *t*-statistic for the strength of correlation in the observed dataset was compared against 10,000 datasets with randomized assignments between promoters and RefSeqs. The value of *t*-statistic for observed data (54.29404) was compared with *t*-statistics for 10,000 randomized datasets (mean − 0.00959) and the *P* value obtained was lesser than 2.2e-16.

Might the correlation between expression breadth and the number of Tfbs be an artifact owing to a correlation with a further parameter? Might indeed the chromatin status or underlying nucleotide content be alternative and better predictors? To explore this we consider a multiway set of correlations and partial correlations, that is each variable predicting breadth, controlling for all others (Table 4, see also Figure 4). This suggested a link between BoE and the number of transcription factor binding sites to be the strongest correlation (*rho* = 0.48, Figure 4 and Table 4), even after controlling for all other parameters (the corresponding partial correlation in Table 4 has *rho* = 0.40). While the raw data show some scatter (Figure 5a-c) the monotonic trend is easily visualized in a box plot based on deciles of the data by BoE (Figure 5e).

**Table 4:** **Correlations and partial correlations**

		**BoE** ^**a**^	**BoE-partial**	**Average** ^**a**^	**Average partial**	**Average -conditioned** ^**a**^	**Average conditioned partial**
Parameters	GC	**0.33**	− 0.03	**0.33**	0.00	− **0.07**	0.04
	GC_big	− **0.02**	− 0.04	− **0.00**	− 0.01	**0.01**	− 0.02
	GC3	− **0.08**	− 0.05	− **0.06**	0.05	**0.08**	0.10
	CpG	**0.42**	0.18	**0.42**	− 0.03	− **0.12**	− 0.16
	TfbsNo.	**0.48**	0.40	**0.48**	− 0.06	− **0.13**	− 0.29
	Methyl	− **0.11**	− 0.04	− **0.09**	0.00	**0.04**	0.03
	DNASE1	**0.19**	0.06	**0.20**	− 0.02	− **0.06**	− 0.07
	BoE	**---**	---	**0.94**	0.91	**0.45**	0.63
	Avg^b^	**0.94**	0.91	**---**	---	**0.55**	---^b^
	Avg_cond^b^	**0.45**	0.63	**0.55**	---^b^	**---**	---

Partial correlations (signified by the *-*partial suffix) are Spearman correlations between the column variable with each raw variable, that is, parameter or explanatory variable, controlling simultaneously for all other parameters.

The parameters include four measures of GC-content: GC-content in a 1 kb proximal promoter (GC), GC-content in a 20 kbps window around the promoter (GC_big), GC-content in a third codon position (GC3), frequency of CpG sites (CpG). TfbsNo. describes the number of transcription factor binding sites in the promoter. Methyl is a measure of methylation while DNASE1 is the signature of open-chromatin.

^a^Straight correlations.

^b^When calculating partial correlations for each measure of average expression (*i.e.* average expression and average-conditioned-on-breadth), we omitted the other measure of average expression.

As regards possible chromatin effects we observe (Table 4 and Figure 4), as expected, a positive correlation between BoE and ENCODE DNASE1 signal (Spearman’s *rho* = 0.19, *P* value <2.2e-16), and a negative correlation between BoE and ENCODE methylation signal (Spearman’s *rho* = – 0.11, *P* value <2.2e-16). There was also a strong correlation of BoE with GC- and CpG-content (*rho* = 0.33, *P* value <2.2e-16; and *rho* = 0.42, *P* value <2.2e-16, respectively). There was also a strong correlation between CpG and TfbsNo. (*rho* = 0.45, *P* value <2.2e-16, Figure 4) and GC-content and the number of Tfb sites (*rho* = 0.29, *P* value <2.2e-16, Figure 4). Strikingly, however, on multiway partial correlation, the strength of these effects tended to diminish dramatically. Correlation with GC went from a raw correlation of 0.33 to a partial of just 0.03. Correlation with DNASE1 went from 0.19 to just 0.06 and the methyl effect diminished from − 0.11 to just − 0.04. By contrast the effect of transcription factor number was relatively unchanged (0.48 prior to multiway analysis, 0.4 after). These results suggest that the chromatin effects may mediate the control of gene expression, but the prediction of BoE is best done via transcription factor information (and this is most likely the casual association).

Closer scrutiny of the impact of GC content as a predictor supports the view that it is GC of the core promoter rather than a more regionalized GC content that impacts BoE. When we divided promoters into low GC (less than 50%, n = 5,650) and high GC (more or equal than 50%, n = 25,710), the second group had more than three times higher average BoE (the exact ratio was 0.3379/0.099 = 3.41) and on average bound more than four times more TFs (9.55/2.29 = 4.17). The GC content of proximal promoters (defined as a 1 kbps window) was much higher than that of surrounding DNA sequences (20 kbps window): 0.594 *versus* 0.463 (Welch Two Sample *t*-test, *P* value <2.2e-16). Similar results were reported by the ENCODE consortium who found that GC content of ChIP-seq sites was 61 ± 5% for TSS-proximal peaks [29]. The exact cause of this effect is not yet fully understood. Although some TF motifs are GC-rich [30], these are usually much smaller than the actual ChIP-seq peaks (8 to 21 bps *vs.* approximately 250 bps).

As shown in Table 5, including GC-content-related measures (that is, GC content, CpG, and CpG*oe*) in a support vector machine (SVM) learning dataset does not substantially increase prediction accuracy over the simple SVM trained with data on Tfbs numbers. (CpG*oe* is a measure of observed CpG frequency normalized by the frequencies of G and C nucleotides proposed to work as a proxy of methylation over large evolutionary timescales [17]). Nevertheless, partial correlation between BoE and CpG persisted after controlling exclusively for TfbsNo. (*rho* = 0.214). However, partial correlation between BoE and TfbsNo. was higher, after controlling exclusively for CpG (*rho =* 0.37), suggesting this effect was dominant. Taken together, these results suggested that CpG was more of a place marker than a key part of the mechanism. Promoter GC content was clearly distinct from the isochore GC content or GC3 (while the latter two correlated closely together, see Additional file 1: Figure S7).

**Table 5:** **SVM trained with data on the numbers of interacting Tfbs (SVM-Tfbs) improved on simple correlation, but adding data on GC content (SVM-Tfbs + GC) did not lead to further improvement of predictions**

	**Correlation**	**SVM-Tfbs**	**SVM-Tfbs + GC**
T	**0.447**	**0.6265**/0.1794/0.9329	**0.6328**/0.1351/0.9368
PC	**0.53**	**0.6791**/0.2493/0.934	**0.6761**/0.2614/0.9354
CCL	**0.61**	**0.7460**/0.2541/0.9447	**0.7474**/0.2874/0.9432

For SVM-Tfbs and SVM-Tfbs + GC three correlations were given: prediction (results in bold), scrambled (response vector was randomized when learning -- this is a negative control), and retained (response vector was retained in the learning dataset -- this is a positive control). SVM-Tfbs was trained with data on the numbers of interacting Tfbs only. SVM-Tfbs + GC training dataset additionally included data on promoter GC and CpG content.

We note that we see little or no evidence for a class of genes so highly broadly expressed that they dispense with TFs altogether. In fact, there were only 39 broadly expressed genes with fewer than 10 high-quality TFs in a broad 10 kb window around the TSS (Additional file 2: Table S1).

### Expression level is not well predicted by Tfbs number

The correlation between the number of transcription factor binding sites and the BoE was strongest at the cutoff for a gene to be ‘on’ set at 10 TPM. The correlation was much weaker at the cutoff of 100 TPM, and disappeared at the cutoff of 1,000 TPM (Table 6). One interpretation of this result is that TFs control mostly where the gene is expressed, but not at what level. The strength of expression might be regulated predominantly by higher-level chromatin architecture or epigenetic marks. To address this in more detail, we also ask whether Tfbs number predicts the level of expression of a gene.

**Table 6:** **The number of transcription factor binding sites correlated with the BoE, the mean expression, and the median expression, but not with the value of the maximum expression of a transcript**

**Expression feature**	**The strength of correlation Tfbs No.**	***t*** ^**a**^	***df*** ^**b**^	***P*** **value** ^**c**^
Breadth at the cutoff of 10 TPM	*r*_*p*_ = 0.448	*t* = 88.1194	*df* = 30,873	<2.2e-16
Breadth at the cutoff of 100 TPM	*r*_*p*_ = 0.16	*t* = 28.6497	*df* = 30,873	<2.2e-16
Breadth at the cutoff of 1,000 TPM	*r*_*p*_ = 0.035	*t* = 6.1749	*df* = 30,873	6.70E-10
Mean expression	*r*_*p*_ = 0.13	*t* = 23.3451	*df* = 30,873	<2.2e-16
Median expression	*r*_*p*_ = 0.254	*t* = 46.2675	*df* = 30,873	<2.2e-16
Maximum expression	*r*_*p*_ = − 0.02	*t* = − 3.5161	*df* = 30,873	0.00043
Breadth-conditioned mean expression	*r*_*p*_ = −*0.041*	*t* = − *6.4983*	*df = 25,040*	8.277e-11
Breadth-conditioned median expression	*r*_*p*_ = − *0.026*	*t* = − *4.1194*	*df = 25,040*	3.811e-05

Here the mean/median were defined across all samples even if the expression level was zero. As this forces a necessary correlation with breadth, we repeated the same using mean/median defined only for samples where expression is seen (breadth-conditioned mean and median expression).

TPM stands for ‘tags per million’. The TPM value of 10 was accepted as the standard threshold for a gene to be ‘on’ in a given library. The BoE was the fraction of samples in which the gene was ‘on’. 10 TPM corresponded to approximately 3 mRNA copies per cell based on 300,000 mRNAs/cell [28].

^a^ *t*-statistic.

^b^Degrees of freedom.

^c^Number of data-points.

Previous authors suggested a strong correlation between the BoE and average expression of a transcript [17]. However, this might be, at least partially, a methodological circularity. If one permits all genes that are unexpressed in a given tissue to score zero for that tissue (definition 1), then tissue specific genes ‘mean expression’ will be dominated by the sum of zeros, hence forcing a tissue specific genes to have low mean level. When instead, we define mean level, as the mean level of expression, in the tissues within which the gene is expressed (definition 2), we find no evidence for a correlation between expression breadth and expression levels in FANTOM5 using parametric statistics, and only weak evidence using non-parametric statistics (Table 7).

**Table 7:** **There is evidence for a real correlation between expression breadth and expression levels if non-parametric statistics are used**

**Mean** ***vs.*** **breadth (Pearson correlation) parametric statistics**	**Mean-conditioned-by-breadth** ***vs.*** **breadth (Pearson correlation) parametric statistics**	**Mean** ***vs.*** **breadth (Spearman correlation) non-parametric statistics**	**Mean-conditioned-by-breadth** ***vs.*** **breadth (Spearman correlation) non-parametric statistics**	
***r***_***p***_ = **0.33**, p <2.2e-16, T	***r***_***p***_ = − **0.012**, p = 0.0546, T	***rho*** **= 0.94**, p <2.2e-16, T	***rho*** **= 0.45**, p <2.2e-16, T	10 TPM
***r***_***p***_ = **0.26**, p <2.2e-16, PC	***r***_***p***_ = **0.03**, p = 4.486e-09, PC	***rho*** **= 0.95**, p <2.2e-16, PC	***rho*** **= 0.5**, p <2.2e-16, PC	
***r***_***p***_ = **0.34**, p <2.2e-16, CCL	***r***_***p***_ = **0.12**, p <2.2e-16, CCL	***rho*** **= 0.957**, p <2.2e-16, CCL	***rho*** **= 0.44**, p <2.2e-16, CCL	
***r***_***p***_ = **0.64**, p <2.2e-16, T	***r***_***p***_ = − **0.00015**, p = 0.9892, T	***rho*** **= 0.6**, p <2.2e-16, T	***rho*** **= 0.41**, p <2.2e-16, T	100 TPM
***r***_***p***_ = **0.52**, p <2.2e-16, PC	***r***_***p***_ = **0.07**, p = 4.264e-12, PC	***rho*** **= 0.66**, p <2.2e-16, PC	***rho*** **= 0.49**, p <2.2e-16, PC	
***r***_***p***_ = **0.66**, p <2.2e-16, CCL	***r***_***p***_ = **0.22**, p <2.2e-16, CCL	***rho*** **= 0.66**, p <2.2e-16, CCL	***rho*** **= 0.38**, p <2.2e-16, CCL	
***r***_***p***_ = **0.76**, p <2.2e-16, T	***r***_***p***_ = − **0.027**, p = 0.4422, T	***rho*** **= 0.24**, p <2.2e-16, T	***rho*** **= 0.32**, p <2.2e-16, T	1,000 TPM
***r***_***p***_ = **0.82**, p <2.2e-16, PC	***r***_***p***_ = **0.021**, p = 0.4608, PC	***rho*** **= 0.28**, p <2.2e-16, PC	***rho*** **= 0.47**, p <2.2e-16, PC	
***r***_***p***_ = **0.88**, p <2.2e-16, CCL	***r***_***p***_ = **0.12**, p = 0.00016, CCL	***rho*** **= 0.25**, p <2.2e-16, CCL	***rho*** **= 0.34**, p <2.2e-16, CCL	

Mean-conditioned-by-breadth is the mean where the average signal is calculated only in tissues in which the gene was ‘on’.

Results obtained using non-parametric statistics are likely to be correct, as the distributions of both BoE and mean expression are not normal.

T = tissue samples, PC = primary cell lines, CCL = cancer cell lines.

**Figure 6:**
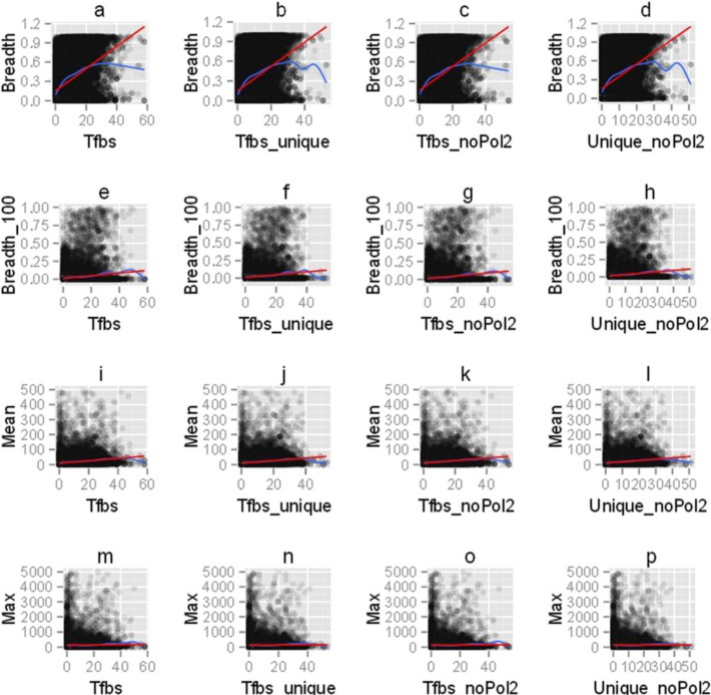
**The correlation between the BoE in human tissues, the mean and the maximum expression, and the number of transcription factor binding sites.** This figure consists of 16 parts identified as **(a - p)**. Four measures related to the BoE were considered: **(a, b, c, d)** the BoE at the cutoff of 10 TPM, **(e, f, g, h)** the BoE at the cutoff of 100 TPM, **(i, j, k, l)** the mean expression, and **(m, n, o, p)** the maximum expression. The number of transcription factor binding sites was estimated in four different approaches: **(a, e, i, m)** the total number, **(b, f, j, n)** the number of unique binding sites, **(c, g, k, o)** the total number excluding RNA polymerase II binding sites, and **(d, h, l, p)** the number of unique binding sites excluding the polymerase. The red line signified the linear model for the smoother line, while the blue line signified the non-linear model. The correlation between the number of transcription factor binding sites and the BoE at the cutoff of 10 TPM was robust under four different approaches to estimating the number of transcription factor binding sites. Interestingly, this correlation was driven by transcripts with between zero to 20 binding sites (*r*_*p*_ = 0.42), and was much weaker for promoters with more than 20 sites (*r*_*p*_ = 0.098). At the value of approximately 20 on the X-axis **(a-d)**, the blue smoother (the non-linear model) reached a plateau and diverged from the red smoother (the linear model). This figure suggests that the correlation presented here was strongest at the cutoff for the BoE of 10 TPM, and was not biased by the polymerase or another individual transcription factor. The correlations with the mean expression were likely secondary to the correlation with the BoE (see Results: Broadly expressed genes have more transcription factor binding sites).

We can ask how these two definitions also relate to Tfbs number. Using definition 1 of mean/median expression, we find that the number of transcription factor binding sites correlates with the mean expression, and the median expression, but not the maximum expression of a transcript (see Table 6, Figure 6). However, the correlations become very weak (with mean: *rho* = − 0.056, *P* value = 8.585e-16; and with median: *rho* = − 0.0151, *P* value = 0.0309), when they were calculated only across tissues in which the gene was ‘on’ (at the cutoff of 10 TPM). As definition 1 forces the mean and median across all tissues to co-vary with the breadth, the strong correlations found using definition 1 were most likely just detecting the primary underlying correlation with the BoE. We conclude that the Tfbs number is a poor predictor of expression rates when expression breadth is not a compounding factor.

### The correlation between TF binding sites and expression breadth is robust

The above results strongly support the view that more TF binding is correlated with expression in more tissues. How robust is this result? Is it true in both normal and diseased states? Is it robust to control for whether or not RNA PolII is included in the set of binders? Is it dependent on the assumed size of the promoter? In the three sections below we consider these and other possible confounders.

### Correlations are robust to alternative assumptions of the promoter size

Given the decay in the rate of the increase of the number of TFs as the size of promoters expands, we presume that increasing the assumed promoter size should start to cause a decay in the correlation between expression and the number of TFs, just because we are diluting signal (true TF binding) with noise (spurious or unassociated binding). As expected (Figure 2c), the correlation between the BoE and the number of transcription factor binding sites, although robust to the variation in window size, decreases as the size of the analysis window increases. A converse interpretation of this is that Tfbs controlling the BoE are enriched close to the transcription start sites.

The inter-relationship, while strongest for small window sizes, persisted for windows up to 40 kb in size (Figure 2c), well beyond the 6 kb limit of effective promoter size. We expect this limit to be greater than that derived from the rate of increase of TFs measure (circa 3 kb ± TSS) as it takes a considerable dilution of the signal of the TF loaded TSS to remove any correlation. The trend was similar when the ENCODE meta dataset from the 2011 freeze [25] was compared with the broader 2012 freeze [26]. Both these datasets were comprehensive in their coverage of transcription factors (148 and 161, respectively). Both data freezes also covered a wide sample space: the earlier freeze with 71 cell types and 24 additional experimental cell culture conditions [31], and the later freeze with 91 human cell types with various treatments [32]. Finally, the trends detected were robust to alterations in the quality cutoff for ENCODE transcription factor binding sites (Figure 2a-c).

### The correlations are stronger across cell lines than across gross tissues

To control for the possibility of a sample bias or differences between normal and diseased tissues, we tested whether the correlation between the BoE and the number of transcription factor binding sites held across the entire FANTOM5 sample space. Figure 5a and Figure 6 show the results for human tissues. We confirmed that the trends are also seen for primary cells (Figure 5b and Additional file 3: Figure S1) and cancer cell lines (Figure 5c and Additional file 4: Figure S2). The Pearson correlation coefficient (*r*_*p*_) between the BoE and the number of transcription factor binding sites equaled 0.53 for primary cells, and 0.61 for cancer cell lines. The correlation is strikingly stronger for cell lines than for tissues. It makes sense that the correlation was stronger for primary cells and cell lines (*r*^*2*^ approximately 28% to 37%) than for tissues (*r*^*2*^ approximately 20%), as tissues are complex mixtures of cell types where some of the cell-type-specific signal might have been lost.

### The correlation between the BoE and the number of transcription factor binding sites holds when RNA polymerase II sites are excluded

Above we considered all bindings at promoter regions of genes, including RNA polymerase II binding sites. One might readily object that if one includes PolII binding then highly expressed genes may well have more bindings, if only because they have more PolII. Might then the correlation between the BoE and the number of transcription factor binding sites be driven by RNA polymerase II binding sites, or biased by another abundant transcription factor?

To investigate this we employed four approaches for counting, these being: (1) the total number of binding sites; (2) the number of unique binding sites; (3) the total number of binding sites excluding RNA polymerase II; and (4) the number of unique binding sites excluding the polymerase. We find that the correlation holds regardless of the method (see Figure 6). Indeed, results using these four measures were largely indistinguishable. For example, when polymerase sites were excluded, the correlation between the number of transcription factor binding sites and the BoE in human tissues was 0.434 (t = 84.8393, df = 31,093, *P* value <2.2e-16). The correlation was 0.435 when additionally only unique sites were counted (t = 85.1458, df = 31,093, *P* value <2.2e-16). In comparison, the original correlation including all sites was 0.448 (t = 88.2645, df = 31,093, *P* value <2.2e-16), and when only unique sites were counted 0.45 (t = 88.6194, df = 31,093, *P* value <2.2e-16). Indeed, the three derived measures correlated very highly with the original measure with correlation coefficients in pairwise comparisons of 0.994, 0.997, and 0.992 for unique sites, no PolII sites, and unique sites excluding PolII (all *P* values <2.2e-16), respectively.

We can also turn the data the other way around and ask whether sites with more TF bindings also have more PolII. Such a correlation would provide sound evidence that more TFs do indeed result in more transcription. We find this to be the case. After excluding its own sites, the polymerase signal correlated strongly with the total number of transcription factor binding sites (*r*_*p*_ = 0.75). The correlation between the BoE and the number of transcription factor binding sites persisted after controlling for the polymerase signal (partial Spearman’s correlation coefficient equaled 0.3).

### The divergence of the promoters of paralogs strongly predicts divergence of their expression patterns

As discussed in the introduction, a common method to approach the problem of the degree of promoter-centered control of gene expression has been to ask about the similarity in gene expression of paralogs as a function of the similarity in their promoter domains. In addition to the correlation between the BoE and the number of transcription factor binding sites, we found that the divergence of proximal promoters (measured via a Jaccard Index on Tfbs repertoire – see Materials and methods) correlated strongly with expression divergence, measured by Pearson’s R. The *r*_*p*_ for this trend equaled 0.282 when only the youngest paralog pairs were taken into account. The *r*_*p*_ was even higher when all daughter pairs were taken into account 0.54 (t = 239.8391, df = 136,608, *P* value <2.2e-16). However, the latter comparisons were not fully independent and the results might have been biased by large gene families with a high number of pairwise comparisons. For example, the core histones of H2A@, H2B@, and H3@ families underwent dramatic expansions in placental mammals, resulting in paralogs which are highly co-expressed in proliferating tissues such as the thymus and the testis (manuscript in preparation).

**Figure 7:**
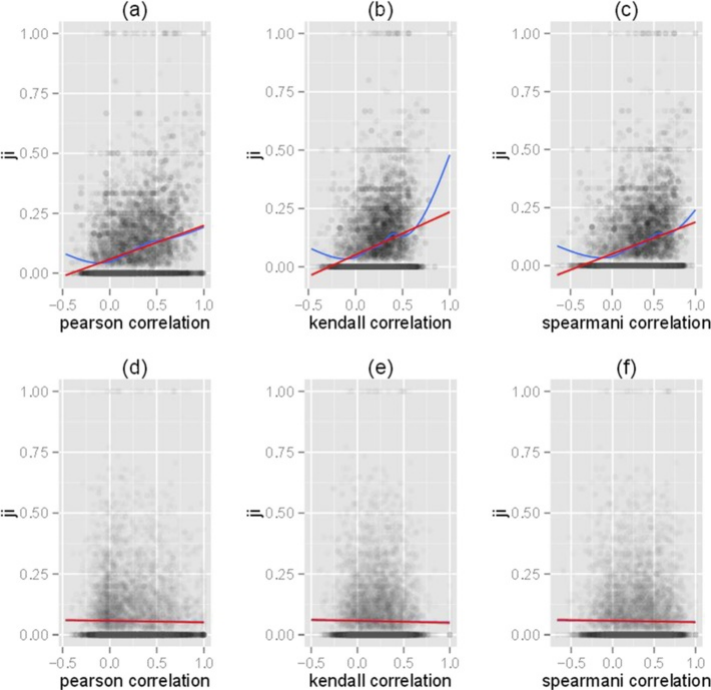
**Expression pattern divergence between paralogs correlated with promoter divergence.** Expression pattern divergence between duplicates was measured with either: **(a)** Pearson’s correlation; **(b)** the Kendall rank correlation coefficient; or **(c)** Spearman’s rank correlation coefficient. Promoter divergence was measured using Jaccard index (JI). The correlation disappeared when duplicate pairs were randomized **(d, e, f)** proving that it was well defined and specific. The red line signified the linear model for the smoother line, while the blue line signified the non-linear model. This figure suggests that the correlation between the BoE and the number of transcription factor binding sites persisted if alternative non-parametric measures of expression distances between paralogs were used. Details of the correlations were given in Table 8.

**Figure 8:**
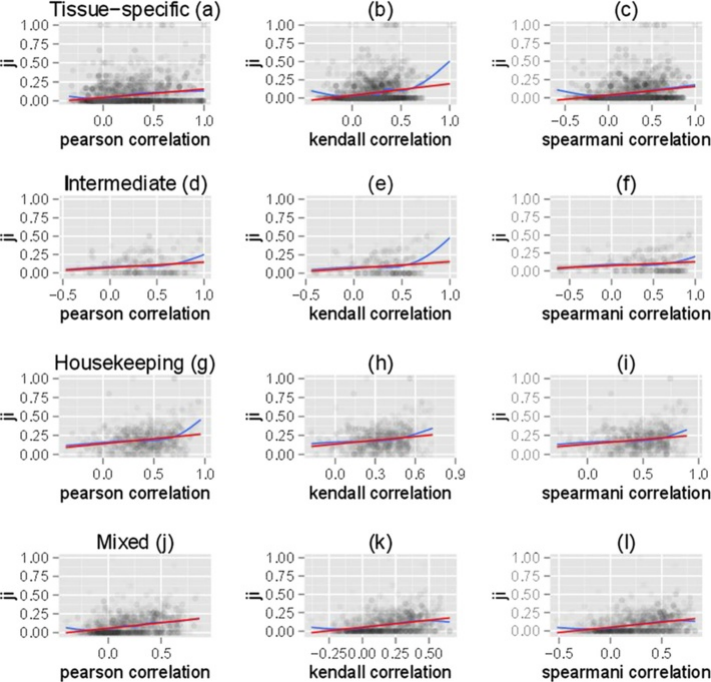
**The correlation between the promoter divergence of paralogs and paralog co-expression was robust in respect to the BoE of target genes.** Four sets of paralog pairs were considered: **(a, b, c)** both paralogs were tissue-specific with the BoE ≤0.33, **(d, e, f)** both genes were intermediate, **(g, h, i)** both genes were housekeeping with the BoE >0.66, and **(j, k, l)** one of the paralogs was tissue-specific and the other housekeeping. Pearson’s **(a, d, g, j)**, the Kendall rank correlation coefficient **(b, e, h, k),** and Spearman”s rank correlation coefficient **(c, f, i, l)** correlations were plotted. The correlation between the Jaccard index (JI) and paralog co-expression was robust under all these conditions. Paralog promoter divergence was measured using the JI. Paralog expression divergence was measured using Pearson’s correlation. The red line signified the linear model for the smoother line, while the blue line signified the non-linear model. Numbers of tags in FANTOM5 were normalized to tags per million (TPM). The TPM value of 10 was chosen as a standard cutoff for a gene to be ‘on’, and the BoE was defined as the fraction of FANTOM5 human tissue samples in which a transcript was ‘on’.

Pearson’s correlation corresponds well to biologist’s intuitive understanding of what co-expressed genes are and has frequently been used in the past to measure expression divergence [1,2,33]. This is because biologists are frequently interested in the identification of tissue-specific or disease-specific biomarkers, and Pearson’s correlation works well for tissues-specific genes. Nonetheless, it is suggested [34] that Pearson’s correlation may be affected by the noise present in microarray data. However, alternative measures such as the Euclidean distance may be biased by normalization [35]. We used three types of correlation to measure paralog co-expression: Pearson’s, the Kendall rank correlation coefficient, and Spearman’s rank correlation coefficient. The correlation between promoter divergence and paralog co-expression held irrespective of the type of the correlation statistic, whether parametric or non-parametric (Figure 7). As expected, the correlation disappeared when duplicate pairs were randomized as a means of negative control (Figure 7d, e, f). The correlations are not simply owing to some paralogs switching from being lowly to broadly expressed (or vice versa). Rather the correlations remains even when we consider paralogs with approximately the same breadth (Table 8, Figure 8).

**Table 8:** **The correlation between the Jaccard index (JI) and paralog co-expression was robust in respect to the BoE**

**The BoE of paralogs**	**Pearson’s correlation between JI and paralog co-expression**	***t*** ^**a**^	***df*** ^**b**^	***n*** ^**c**^	***P*** **value**
Both paralogs were tissue-specific	0.219	16.4535	5,323	8,023	<2.2e-16
Both paralogs were intermediate	0.157	3.7538	551	663	0.000192
Both paralogs were housekeeping	0.217	8.0303	1,297	1,510	2.22E-15
One gene tissue-specific, the other housekeeping	0.324	15.4364	2,026	2,361	<2.2e-16

Transcripts were divided into tissue-specific (BoE ≤0.33), intermediate (0.33 < BoE ≤0.66), and house-keeping (BoE >0.66).

^a^ *t*-statistic.

^b^Degrees of freedom.

^c^Number of data-points.

**Figure 9:**
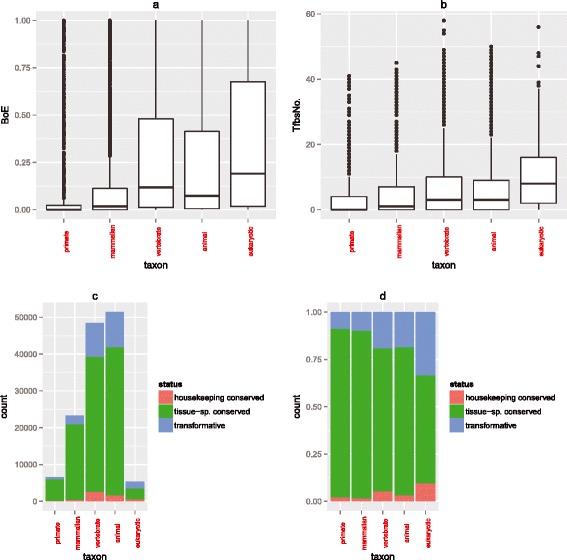
**The correlation between BoE and TfbsNo. is recapitulated over evolution. (a)** A boxplot for BoE depending on age of gene duplication. **(b)** A similar boxplot of TfbsNo. Young genes are more tissue-specific and have fewer TF binders. The correlation between TfbsNo. and BoE was strongest for young genes (Spearman *rho* = 0.531, 0.513, 0.46, 0.447, and 0.403 for increasingly older taxon groups, from primate through to eukaryotic genes). To explain the origins of additional tissue-specific genes in younger taxa, we divided duplication events into three subclasses: ‘housekeeping conserved’, ‘tissue − sp. conserved’, and ‘transformative’. **(c, d)** barplots for the three different mechanistic subtypes of gene duplication events (absolute numbers and relative proportions thereof, respectively).

While the divergence of expression between paralogs is predicted by the divergence of transcription factor repertoire, we additionally observe a trend for young duplicates to be preferentially tissue-specific and have fewer transcription factor binding sites in their promoters. Duplicates mapping to the youngest taxa group (that is, primates) have average BoE almost four times lower, and average TfbsNo. 2.7 times lower (Figure 9a, Tables 9 and 10) than duplicates mapping to the oldest group (that is, eukaryotic). Genes that originated though mammalian gene duplication events had intermediate BoE, at approximately 155% of primate BoE, and less than half of the average eukaryotic BoE and TfbsNo. The differences in mean BoE and TfbsNo, were highly statistically significant with all pairwise comparisons having very low *P* values (see Additional file 5: Table S3 and Additional file 6: Table S4).

**Table 9:** **Young duplicates were more tissue-specific**

	**Taxa group**	**Mean BoE**	**sd**	**n**
1	Primate	0.09	0.22	2,359
2	Mammalian	0.14	0.27	3,783
3	Vertebrate	0.28	0.32	14,518
4	Animal	0.24	0.31	12,329
5	Eukaryotic	0.35	0.36	1,757

Each taxon group excludes duplications mapping to taxa of preceding groups. For example, the vertebrate group consists of vertebrate duplications which are not mammalian. All pairwise comparisons were significantly different with very low *P* values (see Additional file 5: Table S3).

**Table 10:** **Young duplicates had fewer Tfbs regulators**

	**Taxa group**	**sd**	**n**	**TfbsNo.**
1	Primate	6.86	2,034	3.66
2	Mammalian	7.51	3,647	4.81
3	Vertebrate	8.13	14,322	6.60
4	Animal	7.74	12,124	6.02
5	Eukaryotic	9.43	1,728	10.02

Each taxon group excludes duplications mapping to taxa of preceding groups. For example, the vertebrate group consists of vertebrate duplications which are not mammalian. All pairwise comparisons were significantly different with very low *P* values (see Additional file 6: Table S4).

To investigate the origin of new tissue-specific genes, we divided duplication events into three subclasses: ‘housekeeping conserved’ (both paralogs were housekeeping), ‘tissue − sp. conserved’ (both paralogs were tissue-specific), and ‘transformative’ (where one daughter gene was housekeeping while the other was tissue-specific). The relative proportion of ‘tissue − sp. conserved’ events increases for younger taxa indicating that this class of duplication events is responsible for the majority of the increase of tissue-specificity observed for young taxa (Figure 9c and d). This accords with a model suggesting that successful duplication events tend to be those with minimal impact [36,37]. It also accords with the finding that tissue-specific genes are more likely to belong to large gene families [2]. The coupling between duplication age and breadth may bias some statistics. If the BoE of a gene in any manner predicts divergence in expression, this bias has the potential to mislead any analysis that considers the degree of divergence between promoters and divergence in expression, as the least diverged duplicates (that is, the youngest duplicates) will be systematically biased towards the tissue-specific end of the spectrum. However, the trend for gradual expression divergence of paralogs was described in both multicellular [1,2], and unicellular organisms [38] where tissue-specificity cannot be an issue.

### Broad expression is associated with specific transcription factors or groups of cooperating factors

**Figure 10:**
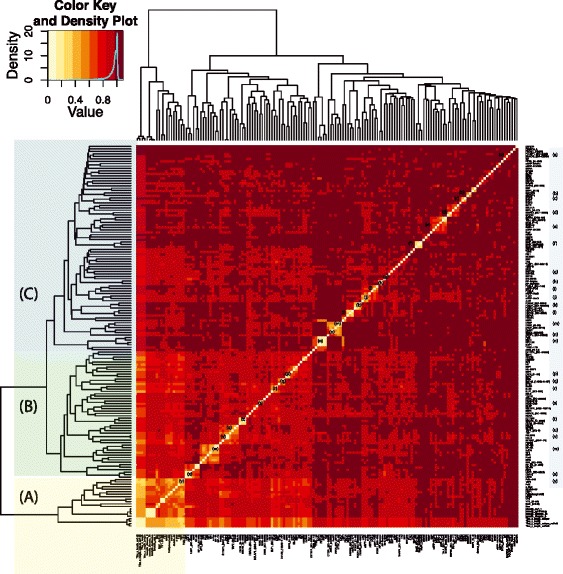
**The clustering of the BoE with the number of transcription factor binding sites.** The BoE clustered with RNA polymerase II and the transcription initiation factor TFIID (TAF1). More interestingly, in human tissues, the BoE also clustered tightly with Mxi1, YY1, NFKB, HEY1, Sin3A, and c–Myc, suggesting these transcription factors were key in determining the BoE (this cluster was marked as A). Other transcription factors formed two clusters with low and high distance to the BoE (these clusters were marked as B and C, respectively). Many clusters of co-localizing transcription factors could be observed and were annotated (a–y). To test the robustness of this analysis, the number of transcription factors was measured in several different ways, which reassuringly clustered together and proved indistinguishable (part of the A cluster). The different measures were: sum of all sites (marked as *Tfbs_x_length*), sum of unique sites (*Tfbs_x_length_unique*), sum of all sites without RNA polymerase II (*Tfbs_x_length_noPol2*), and finally the sum of unique sites without the polymerase (*Tfbs_x_length_unique_noPol2*). The BoE was also transformed in several ways which proved equivalent by forming a tight cluster (part of the A cluster). Namely, the BoE was encoded as either a continuous variable (marked as *breadth_continuous*), discretized into three bins (*breadth_discrete_3*), discretized into 10 bins (*breadth_discrete_10*), or transformed and expressed as a square root (*breadth_sqrt_1*).

The broad-brush correlations that we have addressed above suggest that the more TFs bind a promoter the more broadly expressed the gene. But are there some TFs that are especially influential in driving broad expression or is the effect simply owing to an accumulation of TFs causing increased likelihood of broad expression? To address this, we clustered the BoE with a matrix of transcription factors to identify key associations (Figure 10).

First, the BoE was merged into one matrix with the number of ENCODE transcription factor binding sites. Next, a heatmap was drawn for this matrix in order to determine which transcription factors correlated closest with the BoE, that is, which transcription factors acted as molecular switches for house-keeping expression (Figure 10). The heatmap in Figure 10 uses Pearson’s correlation as the distance measure. Similar results were obtained for human tissues with both the Kendall rank correlation coefficient and Spearman’s rank correlation coefficient (Additional file 7: Figure S5 and Additional file 8: Figure S6). We also investigated distance-based correlations such as Euclidean, Manhattan, or Minkovski distances but these measures did not recover any non-trivial clustering.

**Figure 11:**
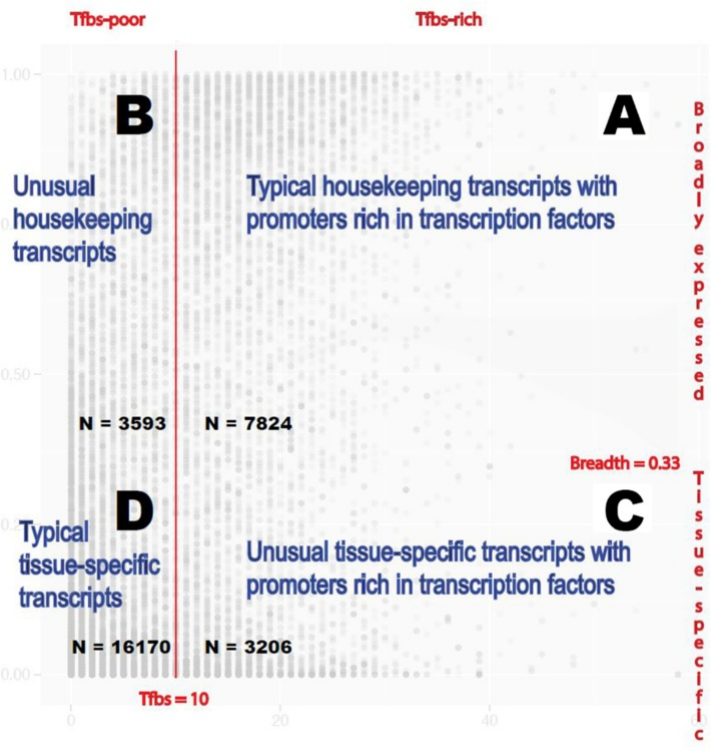
**Conceptual diagram of the four classes of tissue-specific and broadly expressed transcripts rich or poor in transcription factor binding sites.** The four classes were marked with A, B, C, and D. The cutoffs were as follows: 10 transcription factor binding sites for Tfbs-rich, the BoE of 0.33 for broadly expressed, and the TPM value of 10 for a gene to be ‘on’. The BoE was defined as the fraction of FANTOM5 human tissue samples in which a transcript was ‘on’. The biological interpretation of this figure was that typical housekeeping genes were Tfbs-rich, while typical tissue-specific genes were Tfbs-poor. The diagram uses Figure 5a as its background to illustrate the number of transcripts in each of the four classes. The four classes of transcripts will facilitate classification of transcription factors and their impact on individual genes as either activatory or inhibitory (Additional file 9: Table S2).

Four broad classes of transcripts emerged through this integrative analysis. Class A genes are typical broadly expressed Tfbs-rich genes. Class B genes are unusually broadly expressed Tfbs-poor genes. Class C are unusual tissue-specific Tfbs-rich genes, while class D are typical tissue-specific Tfbs-poor genes. These four classes are marked A to D in Figure 11. The numbers of transcripts in A, B, C, and D are 7,824, 3,206, 3,593, and 16,170, respectively. The mean numbers of Tfbs per transcript in each class are: 18.77, 5.41, 16.41, and 1.99 (in FANTOM5 human tissues, 500-cutoff, ENCODE 2011 data-freeze). Here the cutoffs of 10 for Tfbs and 0.33 for the BoE are used. Differentially distributed transcription factors are listed in Additional file 9: Table S2 with their respective frequencies in clusters A, B, C, and D*. P* values were calculated using Fisher’s exact test with a Bonferroni correction for multiple testing.

As expected, the BoE clustered with RNA polymerase II and the transcription initiation factor TFIID – TAF1. This was perhaps unsurprising as the polymerase and TAF1 are constitutive components of the transcription apparatus. More interestingly, in human tissues, the BoE also clustered closely with Mxi1, YY1, NFKB, HEY1, Sin3A, and c–Myc (this cluster was marked with an A in Figure 10) suggesting these transcription factors were among control switches. In human primary cells, the BoE also clustered with the polymerase, TFIID, NFKB, HEY1, Sin3A, and c–Myc (Additional file 10: Figure S3). However, in cancer cell lines, the BoE only clustered with RNA polymerase II and TFIID (Additional file 11: Figure S4) suggesting that cancerous transformation interferes with the majority, except the most rudimentary, control switches for the BoE.

Other transcription factors formed two clusters with either low or high distance to the BoE (these clusters were marked with a B and a C in Figure 10). These two clusters could be enriched in either housekeeping transcription factors (B), or tissue-specific transcription factors (C). In cancer cell lines (Additional file 11: Figure S4), clusters with low (B) and high (C) distance to the BoE could be enriched in oncogenic transcription factors, and anti-oncogenic or tumor-specific transcription factors, respectively.

Many clusters of co-interacting transcription factors can be inferred from Figure 10. These clusters were marked with lowercase letters: (a) Nanog and Pou5f1; (b) Srebp1 and Srebp2; (c) STAT1-3; (d) mef2a and mef2b; (e) GATA-1 and GATA-2; (f) MafF and MafK; (g) BAF170 and BAF155; (h) AP-2 alpha and gamma; (i) FOS and FOSL2; (j) Jun and JunD; (k) FOXA1 and FOXA2; (l) HNF4A and HNF4G; (m) CTCF targeted by three different antibodies; (n) Rad21, SMC3, and CTCFL; (o) Pol3, BRF1, RPC155, and BDP1; (p) E2F1, E2F4, and E2F6; (q) SIX5, Znf143, and ETS1; (r) ELK4 and ELF1; (s) PAX5 targeted by two different antibodies; (t) ZBTB33, BRCA1, and CHD2; (u) NELFe, GTF2B, and TAF7; (v) POU2F2 and Oct-2; (w) NF-YB, NF-YA, c-Fos, and SP1; (x) USF1 and USF2; (y) PolII and TAF1. Some apparent clusters are the same transcription factors targeted by different antibodies (for example, the CTCF rabbit polyclonal, the CTCF_C -20 goat polyclonal, and the CTCF_SC -5916 goat polyclonal antibody) and so should be disregarded. By contrast, some of the clusters are known cooperative complexes. For example, Rad21 and SMC3 form the cohesin complex. The cooperation between Nanog and Pouf1 (alias Oct4) is well described [39]. Other clusters suggest entirely new molecular interactions which should provide material for experimental verification.

### A support vector machine (SVM) method predicts the BoE better than the correlation alone

Given the evidence for cooperativity between TFs in their association with BoE and for key control TFs, we would expect that more sophisticated statistical tools should improve the predictive ability of a model relating TF binding to expression breadth. To address this we established a machine learning approach. Our intention here is not to produce a better statistical model by incorporating non-causal (for example, rate of protein evolution) and causal (TF binding) predictors of expression breadth. Rather we simply wish to ask whether incorporation of the likely causal factors in a more sophisticated statistical framework permits better understanding of the control of expression breadth.

The SVM is a commonly used machine learning approach to prediction. We trained the SVM using a randomly chosen half of the dataset. Each row of the basic SVM training data-frame was a vector representing the number of Tfbs of each gene; although, we also considered SVMs trained using promoter GC and CpG contents (Table 5). The resulting SVM model was then applied to predict the value of the BoE for the other half of the dataset. Note that in this mode of operation with continuous data being predicted, an SVM is best regarded as a form of non-linear regression (rather than a discrete classifier) and hence the appropriate metric for considering its ability is the correlation between the observed and predicted variable (rather than AUC, accuracy, and so on). Such a correlation-based appraisal also permits direct comparison with the simple correlation approach examined above.

As noted above, in cancer cell lines, the correlation between the BoE and the number of transcription factor binding sites is 0.61. The SVM’s prediction improved on this and correlated with the observed value of the BoE with *r*_*p*_ = 0.7460 (Table 5). Results described herein were obtained using the promoter window of 1,000 base pairs but essentially identical values were obtained using the promoter window of 4,000 base pairs (data not shown). As negative and positive controls we used a teaching dataset where the response variable was scrambled or included as one of the training features, respectively. The SVM is thus capable of explaining 58% of the variation, as opposed to the correlation method’s 37%. The prediction accuracy was not due to polymerase signal alone since the SVM performed equally well when TAF1 and all types of polymerase II sites were removed (*r*_*p*_ = 0.759). Moreover, the predictor did not simply rely on summing up of Tfbs, since an SVM trained using only the sums of Tfbs as features (*Tfbs_x_length*, *Tfbs_x_length_unique*, *Tfbs_x_length_noPol2*, *Tfbs_x_length_unique_noPol2*) did not improve on the simple correlation (*r*_*p*_ = 0.646, *r*^*2*^ = 0.42). The number of support vectors was high (approximately 60% of the training cases) suggesting that no simple discriminatory features could be found. Taken together, these results underlined a cooperative effect of many Tfbs acting together and clustering in a narrow window around the TSS to control the BoE.

### Are promoters in open chromatin ‘sticky’?

Even though specific interactions can be identified, that a simple correlation approach can capture so much is striking. The fact that a correlation approach works is consistent with the notion that most transcription factors in eukaryotes are activators. There is, however, an alternative interpretation of the correlation between number of TFs and increasing expression, this being that it reflects more a passive process of spurious TF binding. A simple model in which open/transcribed chromatin is to some degree ‘sticky’ could in principle predict the same correlation. At the limit there might be a single TF that forces broad expression, but because of its presence other TFs are recruited, not because they are needed, but because TFs might be attracted to open chromatin and transcriptional hotspots as iron filings are attracted to a magnet. In this context, were for example GC rich sequence ‘sticky’ for transcription factors, this might explain the GC-TF correlations. However, such a ‘sticky’ model would require strong binding of the TFs to the DNA, rather than weak and short-lived non-specific interactions which are most unlikely to resist the processing of the TF-DNA interaction in ChIP-seq methodology.

Several facts argue against the ‘sticky’ model. First, our approach has recovered known interacting complexes, such as cohesin and CTCF [40,41], suggesting that much of the signal is owing to functional rather than spurious effects. Furthermore the correlation between breadth and number of transcription factors is more profound for transcripts with fewer than twenty transcription factor binding sites (*r*_*p*_ = 0.42), than it is for transcripts with more than twenty (*r*_*p*_ = 0.098). We would expect the opposite if Tfbs simply accumulated in a runaway positive feedback loop.

**Figure 12:**
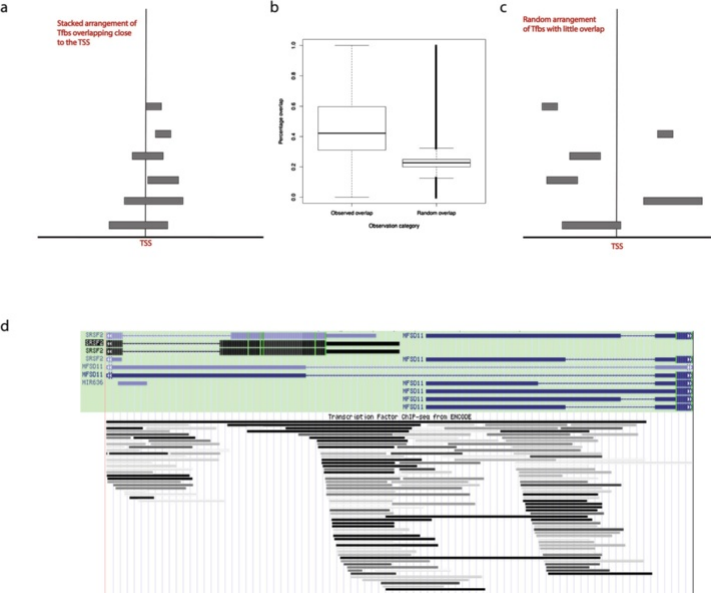
**TF binding sites are clustered (or ‘stacked’) within promoters.** Two theoretically possible alternative Tfbs distributions in proximal promoters are illustrated in **(a)** and **(c)**: **(a)** illustrates a situation where Tfbs are located in a ‘stacked’ arrangement close to the TSS. This arrangement results in high percentage overlap in pairwise Tfbs comparisons. In contrast, **(c)** illustrates a situation where Tfbs are randomly distributed in the proximal promoter. To test for these two trends on the whole genome scale, we calculated percentage overlap in all pairwise Tfbs comparisons in each promoter (1 kb) for 25,930 RefSeq transcripts **(b)**. The observed overlap (corresponding to the ‘stacked’ arrangement of Tfbs illustrated in **(a)** was contrasted against a randomized dataset where Tfbs were assigned random positions within the same proximal promoter (corresponding to the random arrangement of Tfbs illustrated in **(c)** and resulting in a lower percentage overlap in pairwise Tfbs comparisons). As an example, we demonstrate the SRSF2/MFSD11 locus in **(d)** *,* which has the highest number of Tfbs (that is, 71) in our dataset, arranged in the overlapping or ‘stacked’ mode.

In addition, we can ask whether the TF binding sites are clustered within promoters. Were the ‘sticky’ spurious binding model correct, we might also expect that TF bindings sites are randomly located within promoters. By contrast, a model of cooperative binding to DNA and the synergistic mode of action in attracting polymerase and activating transcription, might predict TF bindings sites to cluster and overlap more than expected by chance. These two alternative concepts are illustrated in Figure 12a and c. Figure 12a illustrates a situation where Tfbs are located in a ‘stacked’ arrangement close to the TSS. This arrangement results in high percentage overlap in pairwise Tfbs comparisons. To test for any trend on the whole genome scale, we calculated percentage overlap in all pairwise Tfbs comparisons in each promoter (1 kb) for 25,930 RefSeq transcripts (Figure 12b). The observed overlap (corresponding to the ‘stacked’ arrangement of Tfbs illustrated in Figure 12a) was contrasted against a randomized dataset where Tfbs were assigned random positions within the same proximal promoter (corresponding to the random arrangement of Tfbs illustrated in Figure 12c and resulting in a lower percentage overlap in pairwise Tfbs comparisons). The average observed overlap across all promoters and Tfbs pairs was 0.4586%, much higher than the average overlap in the randomized dataset (0.2295%), suggesting ‘stacked’ rather than dispersed arrangement of Tfbs (*t*-test, *P* value <2.2e-16). These results are in agreement with the general trend demonstrated by ENCODE for almost all transcription factor binding sites to have highest densities very close to the TSS [29,42]. As an example, we show the SRSF2/MFSD11 locus in Figure 12d*.* This locus on chromosome 17 (start at 74,732 kbps, end at 74,734 kbps) has the highest number of Tfbs in our dataset, which are clearly overlapping or ‘stacked’. The SRSF2/MFSD11 locus has 71 Tfbs in the window ± 1,000 bps from the TSS and drives bidirectional transcription of serine/arginine-rich splicing factor 2 (SRSF2) and major facilitator superfamily domain containing 11 (MFSD11).

A further argument against the ‘sticky’ model of Tfbs binding comes from the examination of the BoE of ENCODE Tfbs and their targets. We detected a strong correlation between the average BoE of Tfbs targets and the BoE of respective regulating Tfbs (Additional file 12: Figure S8) with the Spearman *rho* = 0.3176 (*P* value = 0.0001242). Tissue-specific Tfbs bind on average more tissue-specific genes (mean BoE of targets 0.394), than housekeeping Tfbs (mean BoE = 0.468), Wilcoxon rank sum test *P* value = 6.805e-05. Under the pure ‘sticky’ model, we would expect no correlation. The picture is not clear-cut, however. Clearly, tissue-specific transcription factors can have targets in promoters of housekeeping genes. It is possible that housekeeping expression in certain tissue-types demands activation by this tissues unique transcription factors in addition to the standard set of TFs mediating housekeeping expression. Finally, TFs may differ in the degree of ‘stickiness’. Some TFs may be ‘sticky’ towards open chromatin, while others bind very specifically, perhaps playing a key role in the initial act of the opening of the chromatin.

Another argument against the ‘sticky’ model is that open/close chromatin is only a binary signal which cannot account for all the complexities of spatiotemporal gene regulation in vertebrates. Moreover, the correlation between the BoE and DNASE1 sensitivity, an excellent marker of open chromatin, was much weaker than that between the BoE and the number of potential interacting transcription factors (Table 4).

Yet another argument against the sticky model is that TfbsNo. describes the general affinity landscape of promoters for Tfbs binding (that is, the ability to bind TFs). Which TFs are actually bound will depend on a particular tissue and may vary greatly. This is because ENCODE inputs were derived datasets, where individual peaks from different tissues were merged if tagged to the same transcription factor binding site. In fact, counting Tfb sites across different tissues would be a source of a logical error, as a circular association between the BoE and the presence of Tfb sites in multiple tissues would obscure any causal connections.

While multiple lines of evidence argue against the ‘sticky’ chromatin model as the best explanation for the correlation between BoE and number of TF binding sites we cannot entirely refute the hypothesis. Indeed scrutiny of the number of TFs binding the very most broadly expressed genes (right most column in Figure 5e) suggests that these have slightly more TFs than expected given the numbers in the prior bins. There also remains the possibility that the TFs that drive broad expression are themselves ‘sticky’ and attract more TFs. Such a model is more *ad hoc* than the ‘sticky’ chromatin model, having to make the extra assumption that only TFs associated with broad expression are ‘sticky’, for which we see no *a priori* defense. Moreover, this model cannot simply explain some of the above results, such as the stronger correlation for the less broadly expressed genes.

### Prediction of the tissue of expression is moderate at best

Above we have asked if we can predict breadth from knowledge of TFBS. A possibly harder question is whether contained within the promoter architecture is evidence of which tissue or tissues a narrowly expressed gene is expressed in (rather than just the narrowness of that expression). In principle a machine learning approach might be devised to employ the data that we have assembled to tackling such an issue. We took a similar approach to the SVM given above to predict tissues-specific expression (that is, BoE). The main difference was that the continuous response variable was the preferential expression measure (PEM) in a given tissue instead of the BoE.

**Figure 13:**
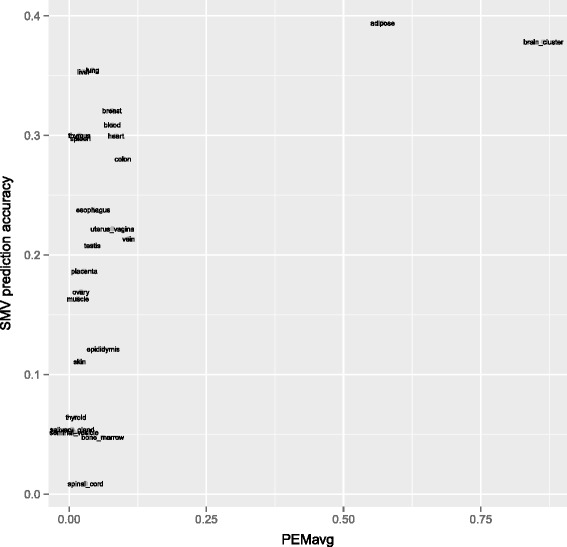
**SVMs predict preferential expression for some tissues, such as the brain and the adipose tissue.** The average preferential expression measure (PEM_*avg*_) expresses the degree of preferential expression of genes expressed in a given tissue. An individual PEM value for each transcript equals its expression in a given tissue divided by its average expression across all tissues. This being a continuous variable, prediction accuracy is the correlation between SVM’s predicted value of the response variable and its observed value in the half of the dataset designated for prediction (the other half was used for training). The highest prediction accuracy was achieved for the adipose tissue and the brain tissue cluster (which were also the two tissues with the highest degree of tissue-specific expression). Overall, there was a strong correlation between the overall degree of preferential expression in a given tissue (PEM_*avg*_) and the power to predict preferential expression in this tissue (Spearman’s correlation of 0.543). For technical details of the SVMs used see Materials and methods.

For a given transcript Y we consider its expression level in tissue X and divide that by Y’s mean expression in all tissues. This ratio is PEM for that transcript in that tissue. So a high PEM for gene Y in tissue X means it is preferentially expressed in tissue X. For all transcripts we then consider the average PEM for a given tissue (PEM_*avg*_). This provides a metric of the degree to which genes are preferentially expressed in tissue X. We then ask about our ability to predict PEM values. To this end we train our SVM against PEM values and ask it then to predict PEM values of genes outside of the training set. If the SVM works, our predictor should correlate with our observed results. Within each tissue we then correlate predicted PEM for all genes against observed PEM for the same genes in the same tissue. These correlation scores are on the Y-axis in Figure 13.

We find that SVMs trained using a matrix of TF frequencies can at best modestly predict preferential expression for some tissues (such as the brain and the adipose tissue, the liver, the lung, and the breast) with the correlation between the predicted and the observed expression of up to *r*_*p*_ = 0.36 for brain (Figure 13) (note that as before the SVM is trained against continuous data so the appropriate metric of accuracy is a correlation coefficient). That brain was the best predicted may well reflect the fact that brain is also the tissue with the highest number of preferentially expressed genes. Indeed, we see an overall correlation between predictive ability (correlation strength) and the mean degree of preferential expression (PEM_*avg*_) for genes expressed in any given tissue (*rho* = 0.54). That is to say, the tissues for which we fail to predict the preferential expression of transcripts are the tissues with few preferentially expressed transcripts (for example, thyroid, salivary gland, skin, bone marrow). Overall, we conclude that at best we have only a moderate ability to predict degree of preferential expression of genes in any given tissue and that this diminishes greatly when the tissues themselves have few tissue specific genes.

### An alternative binary classifier for the prediction of tissue-specific expression

An alternative approach to prediction of tissue-specific (that is, narrow) expression is to divide the transcripts into two categories: tissue-specific and not tissue-specific, at the cutoff of the BoE of 0.33 (that is, one-third of tissues). The approach taken here is identical to the one described above (see: A support vector machine method predicts the BoE better than the correlation alone) except that the input and output measures of the BoE were discretized. Standard validation charts for such a SVM-based binary classifier are shown in Additional file 13: Figure S9. The area under the ROC curve equaled 0.816. Inclusion of GC content does not improve this SVM’s predictive ability, confirming the irrelevance of this feature.

## Discussion

The results above support the view that, to a considerable degree, components of the expression profile, notably expression breadth, and in turn expression breadth divergence, can be predicted from knowledge of the TF binders of a given gene. That such a result was not, for the most part, captured previously, may be explained as limitations in the prior data. That we can recover the BoE result using the previously available Gene Expression Atlas [43], suggests that it is the high resolution ENCODE transcription factor binding data that is key. Given that the ‘sticky’ chromatin model fails to make a parsimonious explanation of the data, we conclude that the data support the suggestion that most eukaryotic TFs are activating.

Here, while touching on expression level, we have concentrated on expression breadth and divergence in breadth. Our results, however, suggest a series of further questions. How, for example, are we to interpret the evidence for cooperation between partners not previously known to be cooperative? Assuming antibody cross-reactivity is not the explanation, these statistically associated TFs suggest experimental tests for apparent cooperativity. Our analysis of whether it is possible to predict the tissue of expression of tissue specific genes suggests that a support vector approach has limited success at best. Given that we have the best success for the tissue (brain) with the most data, it may yet prove to be the case that with better techniques and more data this problem may become more tractable.

A further open issue is whether we can extrapolate our results to non-human species. An ability to infer mutational changes in promoters that are likely to have a major impact on expression, in poorly studied close relatives would be of considerable value in determining those promoters and TFs that might be (or have been) under selection in humans to switch gene expression ‘on’ or ‘off’ in tissues key to human uniqueness. Given the above result (or the moderate ability to predict brain specific genes) there may be some prospect of identifying some lineage specific changes that might have affected human brains.

## Conclusions

We present evidence that expression breadth and paralog expression divergence are strongly predictable with knowledge of transcription factor binding in the proximal promoter. A simple metric, the number of binding transcription factors found on a promoter, is a robust predictor of expression breadth in human tissues. However, prediction of the tissue of expression is moderate at best.

## Materials and methods

### Data sources

#### FANTOM5

Primary FANTOM5 CAGE data [44] (see also: [45]) were processed by the consortium Work-package 4 (WP4) to produce expression tables, mapping CAGE tags to genes, contained in the following three files:

- (T) *human.tissue.hCAGE.hg19.tpm.refgene.osc.txt* - human tissues;
- (PC) *human.primary_cell.hCAGE.hg19.tpm.refgene.osc.txt* - human primary cells;
- (CCL) *human.cell_line.hCAGE.hg19.tpm.refgene.osc.txt* - human cancer cell lines.

These were tab-delimited text files with the header section describing columns, library names, and the total number of reads for each library. These files were pre-processed using standard methods to facilitate their import to the *R* statistical environment with the *read.table* command with row-names being RefSeq accession numbers and column-names identifying tissue, cell-type, or cell line from which CAGE tags were sequenced.

#### ENCODE

ChIP-seq data were described in the section below (Assembling TF binding data to define promoter architecture). The links to the files and description of inputs can be found in the following webpage references [25,26,31,32]. ENCODE Dnase sites, which mark regions of accessible chromatin across the ENCODE set of cell-lines [46,47], and methylation signal for the HeLa cell line were also retrieved from ENCODE [48].

#### Gene Expression Atlas

Gene Expression Atlas (GEA) data [49] were used to independently confirm the correlation between the number of mapping transcription factors and the BoE. We see this as valuable that this strong trend could be detected using two very different expression technologies: one based on next generation sequencing (FANTOM5) and a second one based on microarray hybridization (GEA).

### Assembling TF binding data to define promoter architecture

The 2011 meta dataset included 2,750,490 ChIP-seq peaks for 148 transcription factors, derived from 71 cell-types with 24 additional experimental cell culture conditions [25]. Peaks were called and merged using UCSC clustering tools (*encodeMergeReplicates*, *regClusterBedExpCfg*, and *hgBedsToBedExps*). Crucially, this procedure merges peaks for the same TF across replicates, cell lines, and from different labs into one. Peak scores varied between zero to 1,000 (proportionately to the Tfbs prediction reliability). We used either all data, or only high-quality peaks with the score over half the maximum (that is, 500) to avoid noisy data associated with multi-mapping next-generation sequencing tags. For the correlation between the BoE and the number of transcription factor binding sites, we verified that results analogous to those for the January, 2011, data-freeze [25] were obtained using a broader September, 2012, data-freeze [26]. The 2012 data-freeze consisted of 161 transcription factors and 91 human cell types with various treatment conditions [32]. The ENCODE preprocessing pipeline merges all overlapping binding sites for a TF in different cell lines into a single site. Therefore, the cumulative metric which we calculate corresponds to the total capacity of a promoter to bind TFs (that is, promoter architecture), rather than to the actual number of sites in any particular cell- or tissue-type or across the sample space. This is crucial, as our approach relies on defining the promoter architecture, that is to say the binding landscape, rather than summing up Tfbs across the tissues which would be dangerously tautologous to measuring BoE. However, we took great care not to fall into this trap. On top of the ENCODE’s merging procedure, we employ an additional safeguard by introducing a metric which only counts each TF once even if the promoter can bind this TF at alternative genomic locations (that is, *Tfbs_x_length_unique*). The correlation between *Tfbs_x_length* and *Tfbs_x_length_unique* was almost perfect (*r*_*p*_ = 0.9929) and these two measures supported identical biological conclusions in all analyses (ensuring that no single Tfbs could introduce a bias).

### The Jaccard index for promoter divergence

The JI measured the overlap between two sets of genomic features [50]. The index was calculated as the ratio of the intersection over the union (*JI* = *I*/*U*). JI equaled 1 when the intersection equaled the union (that is when there was a perfect overlap). JI equaled 0 when there was no overlap.

### FANTOM5 gene expression tables

CAGE tags were mapped to RefSeq transcripts ±500 bps from their transcription start sites. The numbers of tags were normalized to tags per million (TPM). Finally, the TPM value of 10 was chosen as the default cutoff for a gene to be ‘on’ (unless stated otherwise). We downloaded RefSeq in BED format from the hg19 UCSC genome browser using the table browser tool. This dataset included 40,856 human transcripts, including messenger RNAs (NM-accession) and non-coding transcripts (NR-accession). Non-coding transcripts included structural RNAs and transcribed pseudogenes. The dataset was later processed with *BEDtools* [51].

### Gene Expression Atlas

Gene Expression Atlas [43] was employed to confirm the correlation between the BoE and the number of transcription factor binding sites in proximal promoters (Figure 5d). Affymetrix average difference (AD) higher that 200 classified a gene as ‘on’ or expressed in a given tissue. Affymetrix ids were mapped to RefSeq ids using *R* annotation object *hgu95aREFSEQ* from the *hgu95a.db* package. ENCODE transcription factors were mapped to RefSeq just as in the FANTOM5 analysis.

### TreeFam and the inference of gene duplications

TreeFam [52] used evolutionary histories of individual genes to construct phylogenetic trees and to date gene duplications [52]. In our hands, TreeFam consistently delivered high quality phylogenetic trees which were congruent with the insights of molecular biologists [53,54].

TreeBeST was the tree-building engine behind TreeFam. TreeBeST merged a maximum likelihood tree from PHYML [55] with neighbor-joining trees based on P-distance, Ka, and Ks. TreeBeST used smart heuristics intended to maximize the similarity between the gene tree and the species tree and to minimize the number of predicted gene duplications and losses.

TreeFam release eight was based on Ensembl version 54 and included 79 species, 1,539,621 genes, and 16,064 families. Phylogenetic timing was used to associate gene duplications with the emergence of different taxa. The Vertebrata and the Bilateria were consistently linked with the most numerous waves of duplications in animals [54]. The algorithm for speciation and duplication inference (SDI) reconciled gene trees with the species dendrogram. SDI also inferred duplications, speciation events, paralogy, and orthology.

### The comparison of all paralog pairs *versus* the comparison of youngest pairs only

Combinatorics explains why there are more possible paralog comparisons than paralogs. In a set of *n* elements, the number of k-combinations was equal to the binomial coefficient (‘*n* choose *k*’). K equals two for pairwise comparisons. For example, there were 10 possible pairwise comparisons in a family of five paralogs. There were 45 legitimate pairwise comparisons in a family of 10 paralogs. However, these comparisons might not be regarded as independent data points, and the results might have been biased by a few large gene families. The best alternative was to perform a comparison between each paralog with only its closest relative (that is, only the most recent duplicate).

### The duplicator

The dataset used in the preparation of this study was released to the public domain as an *R* package called the Duplicator [56]. *R* data-frames with data on duplications mapping to different taxa can be found in *R* helper environments for the package (*env_duplicator_base* and *env_duplicator_vectors*), which are located in the path *duplicator/data/duplicator*. Naming conventions indicate the species of origin. For instance, the *R* data-frames containing human data are called: *dupEvent12_genes_LL_hs* (individual genes) and *dupEvent12_genes_LL_hs2* (paralog pairs).

Data-frames in the package follow the denormalized data model. For example, the *R* data-frame *dupEvent12_genes_LL_hs2* has the following fields: family (TreeFam *family* ID), *node* (unique identifier for each gene duplication node), *taxon* (taxon of duplication), *gene.x* (ENSEMBL ENSG ID for paralog *x*), *familySide.x*, *primary_acc.x* (Entrez IDs for paralog *x*), *gene.y* (ENSEMBL ENSG ID for paralog *y*), *familySide.y*, *primary_acc.y* (Entrez IDs for paralog *y*). *FamilySide* was a flag with values of one or two defining on which side of the duplication node the gene was located; this flag was used to prevent multiple comparisons on the ‘same side’ of the primary duplication node if later duplications occurred.

### The analytical pipeline

The analytical pipeline consisted of three stages: (1) data retrieval and remodeling; (2) the detection of the overlap between promoters and transcription factor binding sites; and (3) parsing, statistical tests, and figure generation.

1. Data retrieval and remodeling (the pre-*BEDtools* stage). This stage consisted of data retrieval from TreeFam and remodeling into *R* data-frames. Helper environments held these data-frames for later use, and stored intermediate results.
2. The detection of the overlap between promoters and transcription factor binding sites (the *BEDtools* stage). We checked if two sets of genomic features overlapped using *BEDtools*. We used mostly *coverageBed* and *intersectBed. CoverageBed* calculates the depth of coverage of features in the file *A* against the file *B*. Herein, *coverageBed* was used to calculate the depth of coverage by ENCODE transcription factor binding sites in promoters (*coverageBed -a ENCODE_Tfbs -b RefSeq_promoters > result*). *IntersectBed* writes the original entry in the file *A* for each overlap when used with the ‘-wa’ option (*intersectBed -a ENCODE_Tfbs -b RefSeq_promoters -wa > result*). This identified the exact type of transcription factor binding sites in the overlap. An alternative analysis pipeline was constructed using *R/BioC* packages *GenomicRanges* and *IRanges*. However, the *R/BioC* pipeline proved prohibitively slow even on SNIC Supercomputers. The alternative pipeline was used only to verify the results of the main *BEDtools*-based pipeline.
3. Parsing, statistical tests, and figure generation (the post-*BEDtools* stage). Postprocessing was performed in *R* and *Bioconductor* (version 2.11). Standard Bioconductor packages such as *Biodist*, *gplot*, *ggplot*, *rtracklayer*, *TxDb.Hsapiens.UCSC.hg19.knownGene*, and *GOstats* were used.

The post-*BEDtools* analysis was divided into the four steps listed below.

Step (1) Parsing *BEDtools* output into an *R* list.
  *BEDtools* output in the browser extensible data (BED) file format was read into *R* as a data-frame. The data-frame was then remodeled into an *R* list called *resJaccard_ENCODE_Tfbs_substrate*. Each element of the list was indexed with a RefSeq transcript id as the accession key. A vector of ENCODE transcription factor binding sites was contained within each element of the list. Transcription factor parsing and creation of the list were performed by the script called *env_bed_jaccard_make_1.R*. Intermediate results were stored in the R helper environment named *env_promoter_bed_500_res_env*.
Step (2) The Jaccard index calculation.
  The JI was calculated using the script *resJaccard_ ENCODE_Tfbs_substrate*. Additionally, a randomized dataset was generated using sampling without replacement. The randomized dataset was used to calculate a control distribution of the JI.
Step (3) Additional calculations.
  The following additional variables were calculated: relative frequencies of different transcription factor binding sites, intersection, union, and the JI depending on the taxon of duplication. These calculations were performed by scripts called *env_jaccard_analyse_result_bed_sampled.R* and *env_jaccard_analyse_random_bed_sampled.R*.
Step (4) Figure generation.
  Manuscript figures were generated automatically using *ggplot2* from intermediate results stored in R helper environments.

### The SVM

The SVM is a commonly used machine learning technique for regression. We used the *R* package *e1071*, an *R* implementation of *libsvm*. Each row of the basic SVM training data-frame was a vector representing the number of Tfbs for each gene; although, we also considered SVMs trained using promoter GC- and CpG-contents (Table 5). The training dataset was scaled and centered. We used a radial kernel as a regression machine. After a grid search for optimal parameter values, the following SVM parameters were used: cost = 1, gamma = 0.01, and epsilon = 0.1. The continuous response variable was either the BoE or the preferential expression measure (PEM).

### Data access

Access to FANTOM5 is provided at the FANTOM5 public website, including the UCSC genome browser mirror, and FANTOM5’s own CAGE-focused ZENBU genome browser.

## Additional files

Additional file 1: Figure S7. Promoter GC-content was a place marker distinct from isochore GC content or GC3. This figure consists of four parts. In the upper-left part, we show that isochore GC-content and GC3 corresponded closely to each other. However, promoter GC-content was quite distinct from the isochore GC-content with a large population of high GC promoters located in low-GC chromosomal regions (the upper-right part). There was no simple direct relationship between methylation and BoE (see bottom-left, the red line is the fitted *loess* curve). Low-GC promoters formed a clearly distinct group from high-GC promoters in terms of average BoE when a correlation between core promoter-GC and BoE was plotted on a scatterplot (bottom-right, the blue line is the fitted *loess* curve).

Additional file 2: Table S1. There is little or no evidence for a class of genes so highly broadly expressed that they dispense with TFs altogether. There were only 39 broadly expressed transcripts (the BoE in human tissues > 0.9) with fewer than 10 TFs in the promoter (defined as a broad 10 kb window around the TSS). Concerted action of multiple TFs appears necessary for housekeeping expression. The columns of the table encode as follows: RefSeq, EntrezID, gene symbol, and gene name.

Additional file 3: Figure S1. The relationship between the BoE, the mean and the maximum expression in human primary cells, and the number of transcription factor binding sites. This figure consists of 16 parts identified as (a *-* p). Four measures related to the BoE were considered: (a, b, c, d) the BoE at the cutoff of 10 TPM, (e, f, g, h) the BoE at the cutoff of 100 TPM, (i, j, k, l) the mean expression, and (m, n, o, p) the maximum expression. The number of transcription factor binding sites was estimated in four different approaches: (a, e, i, m) the total number, (b, f, j, n) the number of unique binding sites, (c, g, k, o) the total number excluding polymerase binding sites, and (d, h, l, p) the number of unique binding sites excluding the polymerase. The red line signified the linear model for the smoother line, while the blue line signified the non-linear model. This figure confirms the robustness of the findings presented in Figure 6 across the FANTOM5 sample space (that is, in human primary cells).

Additional file 4: Figure S2. The relationship between the BoE, the mean and the maximum expression in human cancer cell lines, and the number of transcription factor binding sites. This figure consists of 16 parts identified as (a - p). Four measures related to the BoE were considered: (a, b, c, d) the BoE at the cutoff of 10 TPM, (e, f, g, h) the BoE at the cutoff of 100 TPM, (i, j, k, l) the mean expression, and (m, n, o, p) maximum expression. The number of transcription factor binding sites was estimated in four different approaches: (a, e, i, m) the total number, (b, f, j, n) the number of unique binding sites, (c, g, k, o) the total number excluding polymerase binding sites, and (d, h, l, p) the number of unique binding sites excluding the polymerase. The red line signified the linear model for the smoother line, while the blue line signified the non-linear model. This figure confirms the robustness of the findings presented in Figure 6 across the FANTOM5 sample space (that is, in human cancer cell lines).

Additional file 5: Table S3. *P* values for pairwise BoE comparisons using Wilcoxon rank sum test for data in Table 9. NOTE: *P* value adjustment method: *holm*.

Additional file 6: Table S4. *P* values for pairwise TfbsNo. comparisons using Wilcoxon rank sum test for data in Table 10. NOTE: *P* value adjustment method: *holm*.

Additional file 7: Figure S5. The clustering of the BoE with the number of transcription factor binding sites in human tissues (Kendall rank correlation coefficient). Transcription factors clustering closest with the BoE were marked as A. Other transcription factors formed two clusters with low and high distance to the BoE (these clusters were marked as B and C, respectively). To test the robustness of this analysis, the number of transcription factors was measured in several different ways, which reassuringly clustered together and proved indistinguishable. The different measures were: sum of all sites (marked as *Tfbs_x_length*), sum of unique sites (*Tfbs_x_length_unique*), sum of all sites without RNA polymerase II (*Tfbs_x_length_noPol2*), and finally the sum of unique sites without the polymerase (*Tfbs_x_length_unique_noPol2*). The BoE was also transformed in several ways which proved equivalent by forming a tight cluster. Namely, the BoE was encoded as either a continuous variable (marked as *breadth_continuous*), discretized into three bins (*breadth_discrete_3*), discretized into 10 bins (*breadth_discrete_10*), or transformed and expressed as a square root (*breadth_sqrt_1*).

Additional file 8: Figure S6. The clustering of the BoE with the number of transcription factor binding sites in human tissues (Spearman’s rank correlation coefficient). Transcription factors clustering closest with the BoE were marked as A. Other transcription factors formed two clusters with low and high distance to the BoE (these clusters were marked as B and C, respectively). To test the robustness of this analysis, the number of transcription factors was measured in several different ways, which reassuringly clustered together and proved indistinguishable. The different measures were: sum of all sites (marked as *Tfbs_x_length*), sum of unique sites (*Tfbs_x_length_unique*), sum of all sites without RNA polymerase II (*Tfbs_x_length_noPol2*), and finally the sum of unique sites without the polymerase (*Tfbs_x_length_unique_noPol2*). The BoE was also transformed in several ways which proved equivalent by forming a tight cluster. Namely, the BoE was encoded as either a continuous variable (marked as *breadth_continuous*), discretized into three bins (*breadth_discrete_3*), discretized into 10 bins (*breadth_discrete_10*), or transformed and expressed as a square root (*breadth_sqrt_1*).

Additional file 9: Table S2. Transcription factor frequencies in the four classes of transcripts (A, B, C, D) in respect to the BoE and the number of TFs. Because of the large number of rows, this is a supplementary table available as a text file in the supplementary material. NOTE: The four classes of transcripts were illustrated in Figure 11. Typical broadly expressed Tfbs-rich genes were summarized in column A. Unusual broadly expressed Tfbs-poor genes were summarized in column B. Unusual tissue-specific Tfbs-rich genes were summarized in column C. Typical tissue-specific Tfbs-poor genes were summarized in column D. The numbers of transcripts in respective classes were 7,824, 3,206, 3,593, and 16,170. The frequencies of transcription factors were expressed as fractions of all sites in a given class. The total sums of Tfbs in A, B, C and D were: 146,863, 17,336, 58,978, and 32,134. The mean numbers of Tfbs per a transcript in each class were: 18.77, 5.41, 16.41, and 1.99. *P* values of Fisher’s test were given. All data were calculated for FANTOM5 human tissues. Future work should facilitate classification of all transcription factors and their impact on individual genes as either activatory or inhibitory.

Additional file 10: Figure S3. The clustering of the BoE with the number of transcription factor binding sites in human primary cells. In human primary cells, the BoE clustered with the polymerase, TFIID, NFKB, HEY1, Sin3A, and c–Myc (this cluster was marked as A). Other transcription factors formed two clusters with low and high distance to the BoE (these clusters were marked as B and C, respectively). To test the robustness of this analysis, the number of transcription factors was measured in several different ways, which reassuringly clustered together and proved indistinguishable. The different measures were: sum of all sites (marked as *Tfbs_x_length*), sum of unique sites (*Tfbs_x_length_unique*), sum of all sites without RNA polymerase II (*Tfbs_x_length_noPol2*), and finally the sum of unique sites without the polymerase (*Tfbs_x_length_unique_noPol2*). The BoE was also transformed in several ways which proved equivalent by forming a tight cluster. Namely, the BoE was encoded as either a continuous variable (marked as *breadth_continuous*), discretized into three bins (*breadth_discrete_3*), discretized into 10 bins (*breadth_discrete_10*), or transformed and expressed as a square root (*breadth_sqrt_1*).

Additional file 11: Figure S4. The clustering of the BoE with the number of transcription factor binding sites in human cancer cell lines. In cancer cell lines, the BoE only clustered with RNA polymerase II and TFIID (this cluster was marked as A) suggesting that cancerous transformation disables most normal control switches for the BoE. Other transcription factors formed two clusters with low and high distance to the BoE (these clusters were marked as B and C, respectively). To test the robustness of this analysis, the number of transcription factors was measured in several different ways, which reassuringly clustered together and proved indistinguishable. The different measures were: sum of all sites (marked as *Tfbs_x_length*), sum of unique sites (*Tfbs_x_length_unique*), sum of all sites without RNA polymerase II (*Tfbs_x_length_noPol2*), and finally the sum of unique sites without the polymerase (*Tfbs_x_length_unique_noPol2*). The BoE was also transformed in several ways which proved equivalent by forming a tight cluster. Namely, the BoE was encoded as either a continuous variable (marked as *breadth_continuous*), discretized into three bins (*breadth_discrete_3*), discretized into 10 bins (*breadth_discrete_10*), or transformed and expressed as a square root (*breadth_sqrt_1*).

Additional file 12: Figure S8. There was a positive correlation between the BoE of transcription factors and the average BoE of their targets. The BoE of transcription factors in FANTOM5 tissues was plotted on the X-axis of the scatterplot (signified by *Tfbs_BoE*). The unweighted mean of the BoE of all target genes (that is, all genes that have a given Tfbs in their proximal promoter) was plotted on the Y-axis (signified by *Mean_target_BoE*). As both the independent and dependent variables were highly non-normally distributed, we used non-parametric correlation (Spearman’s *rho* = 0.3176). An alternative measure, a weighted mean, in which that data points were weighted according to the actual number of transcription factor binding sites was also considered and gave almost exactly the same correlation. The blue line is the fitted *loess* curve.

Additional file 13: Figure S9. Performance of SVM models as the predictor of tissue-specific (that is, narrow) expression. Tissue-specific expression was defined as BoE lower than 0.33 (that is, a gene that was expressed in less than one-third of tissues) and transcripts were categorized as either tissue-specific or not (that is, in a binary classification). This figure consists of four panels. The panels display standard predictor validation charts for the basic SVM model (SVM-Tfbs): (a) the ROC curve, (b) the precision/recall graph, (c) the sensitivity/specificity plot, and (d) the lift chart. The curves were averages from 10 different cross-validation runs. The area under the ROC curve equaled 0.816 (standard deviation equaled 0.002325). The parameterization of the curves was performed using the value of the linear SVM output and visualized by printing cutoff values at the corresponding curve positions (the curve was also colored according to the cutoff). The curves were plotted using *R* package *ROCR*. Essentially identical results were obtained a more complex SVM model with added data on GC content (SVM-Tfbs + GC), with AUC = 0.816.
