# Supplementary figures and images for: A simple metric of promoter architecture robustly predicts expression breadth of human genes suggesting that most transcription factors are positive regulators
Source: Genome Biol. 2014 Jul 31;15(7):413. doi: 10.1186/s13059-014-0413-3 (PMC4310617; doi:10.1186/s13059-014-0413-3)

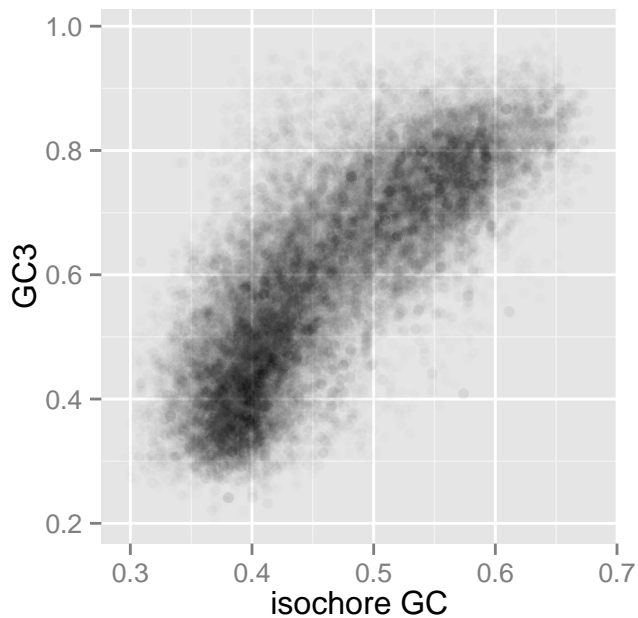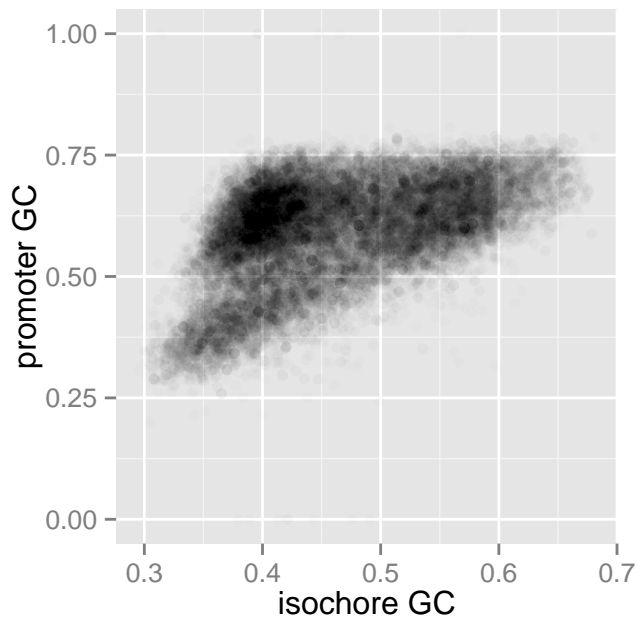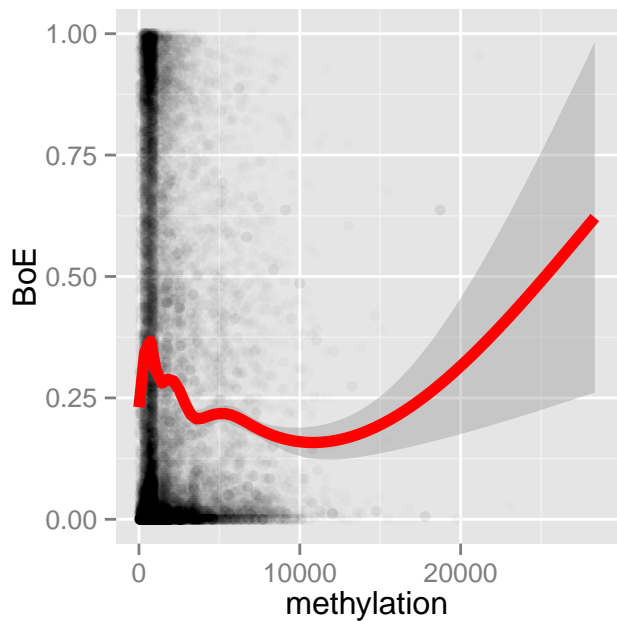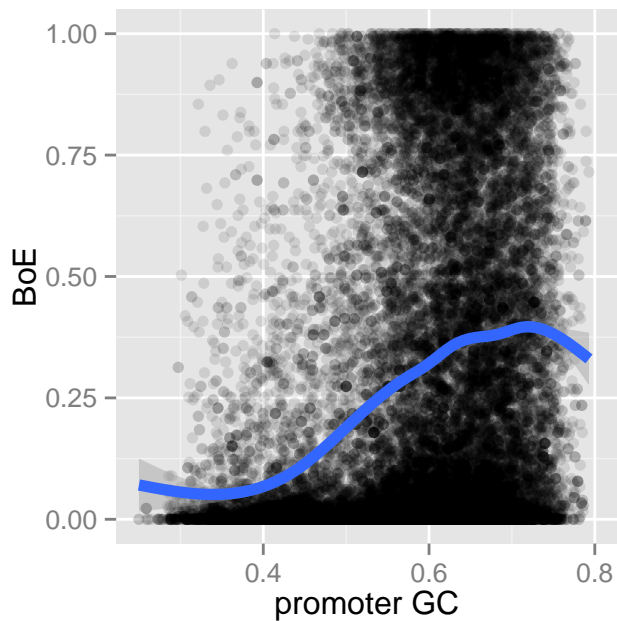

Supplement: Supplementary file 1 — Promoter GC-content was a place marker distinct from isochore GC content or GC3. This figure consists of four parts. In the upper-left part, we show that isochore GC-content and GC3 corresponded closely to each other. However, promoter GC-content was quite distinct from the isochore GC-content with a large population of high GC promoters located in low-GC chromosomal regions (the upper-right part). There was no simple direct relationship between methylation and BoE (see bottom-left, the red line is the fitted loess curve). Low-GC promoters formed a clearly distinct group from high-GC promoters in terms of average BoE when a correlation between core promoter-GC and BoE was plotted on a scatterplot (bottom-right, the blue line is the fitted loess curve). [file 13059_2014_413_MOESM1_ESM.pdf]

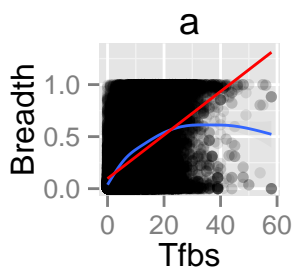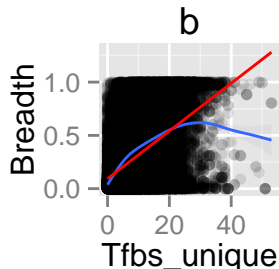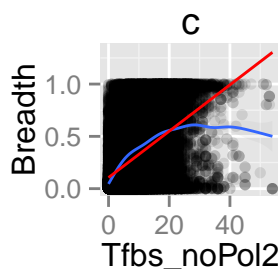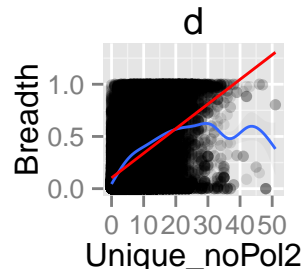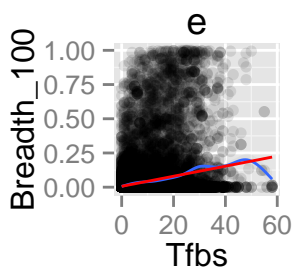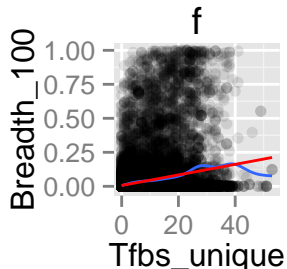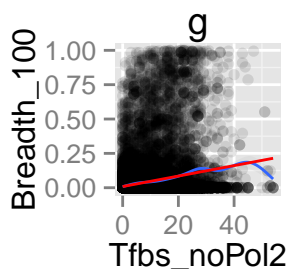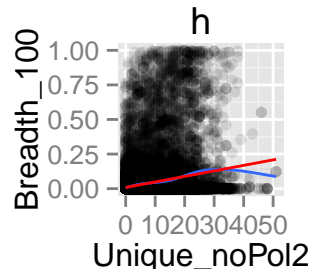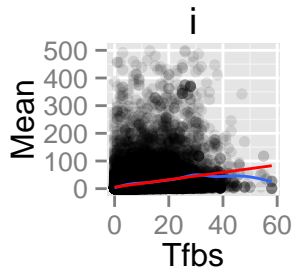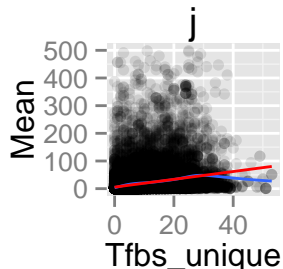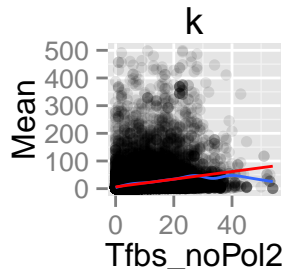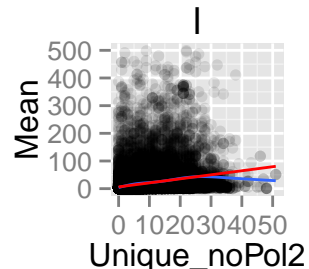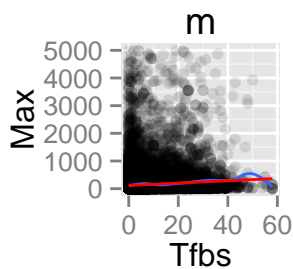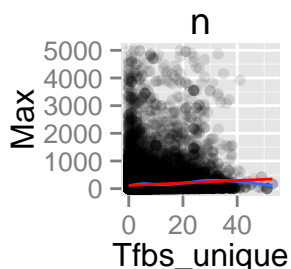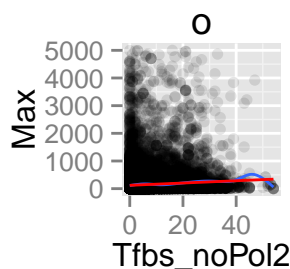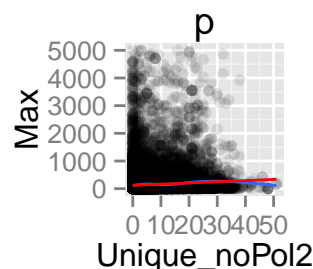

Supplement: Supplementary file 3 — The relationship between the BoE, the mean and the maximum expression in human primary cells, and the number of transcription factor binding sites. This figure consists of 16 parts identified as (a - p). Four measures related to the BoE were considered: (a, b, c, d) the BoE at the cutoff of 10 TPM, (e, f, g, h) the BoE at the cutoff of 100 TPM, (i, j, k, l) the mean expression, and (m, n, o, p) the maximum expression. The number of transcription factor binding sites was estimated in four different approaches: (a, e, i, m) the total number, (b, f, j, n) the number of unique binding sites, (c, g, k, o) the total number excluding polymerase binding sites, and (d, h, l, p) the number of unique binding sites excluding the polymerase. The red line signified the linear model for the smoother line, while the blue line signified the non-linear model. This figure confirms the robustness of the findings presented in Figure 6 across the FANTOM5 sample space (that is, in human primary cells). [file 13059_2014_413_MOESM3_ESM.pdf]

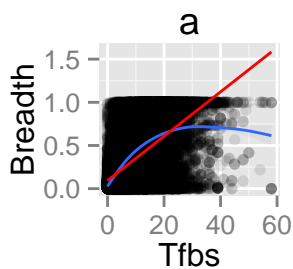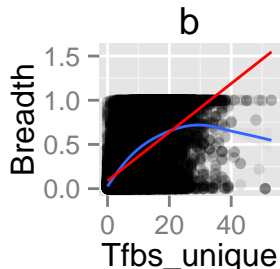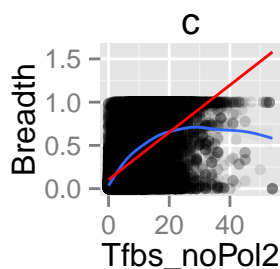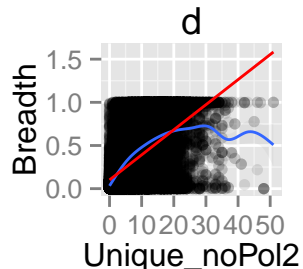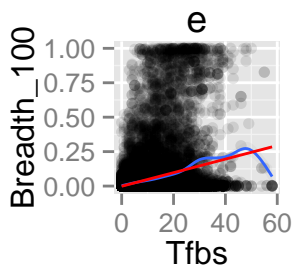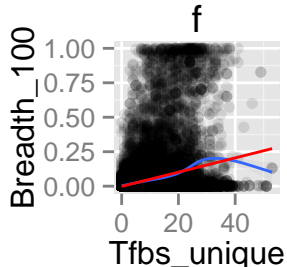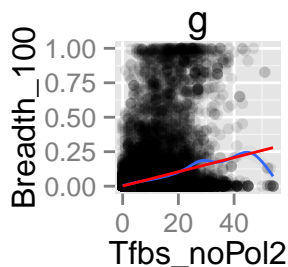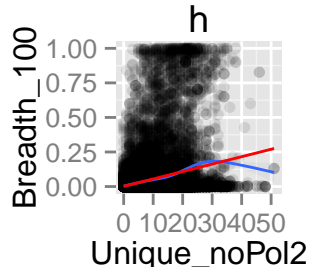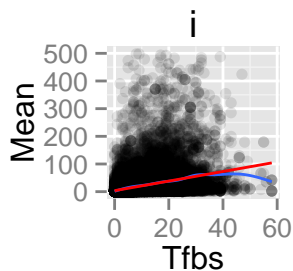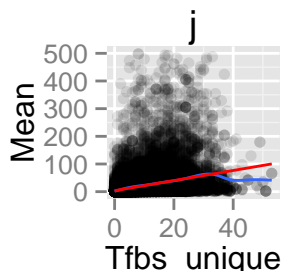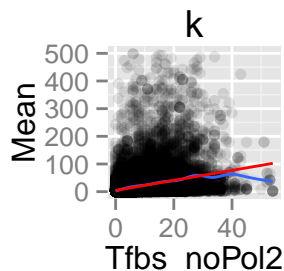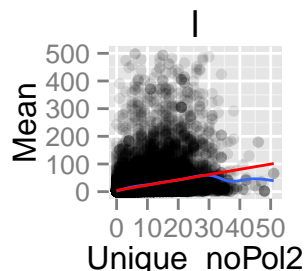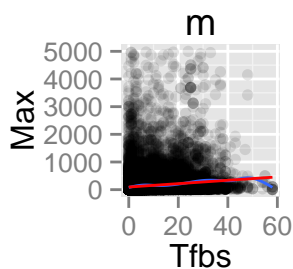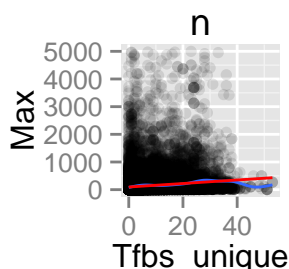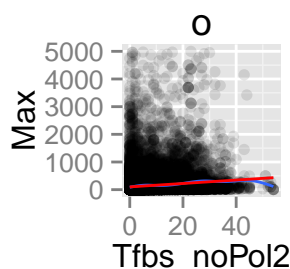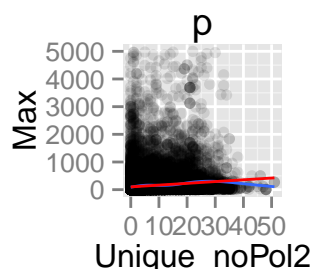

Supplement: Supplementary file 4 — The relationship between the BoE, the mean and the maximum expression in human cancer cell lines, and the number of transcription factor binding sites. This figure consists of 16 parts identified as (a - p). Four measures related to the BoE were considered: (a, b, c, d) the BoE at the cutoff of 10 TPM, (e, f, g, h) the BoE at the cutoff of 100 TPM, (i, j, k, l) the mean expression, and (m, n, o, p) maximum expression. The number of transcription factor binding sites was estimated in four different approaches: (a, e, i, m) the total number, (b, f, j, n) the number of unique binding sites, (c, g, k, o) the total number excluding polymerase binding sites, and (d, h, l, p) the number of unique binding sites excluding the polymerase. The red line signified the linear model for the smoother line, while the blue line signified the non-linear model. This figure confirms the robustness of the findings presented in Figure 6 across the FANTOM5 sample space (that is, in human cancer cell lines). [file 13059_2014_413_MOESM4_ESM.pdf]

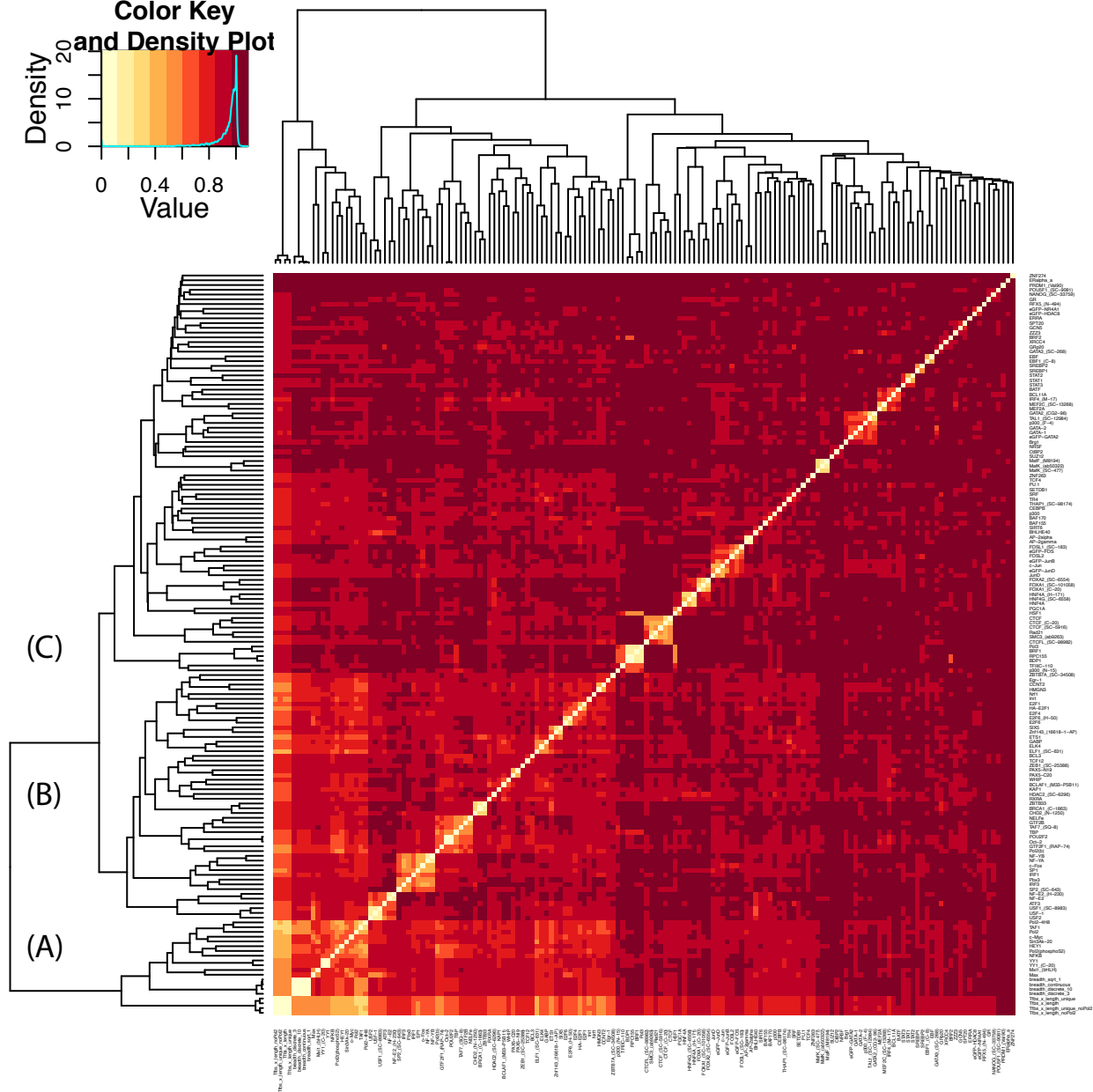

Supplement: Supplementary file 7 — The clustering of the BoE with the number of transcription factor binding sites in human tissues (Kendall rank correlation coefficient). Transcription factors clustering closest with the BoE were marked as A. Other transcription factors formed two clusters with low and high distance to the BoE (these clusters were marked as B and C, respectively). To test the robustness of this analysis, the number of transcription factors was measured in several different ways, which reassuringly clustered together and proved indistinguishable. The different measures were: sum of all sites (marked as Tfbs_x_length), sum of unique sites (Tfbs_x_length_unique), sum of all sites without RNA polymerase II (Tfbs_x_length_noPol2), and finally the sum of unique sites without the polymerase (Tfbs_x_length_unique_noPol2). The BoE was also transformed in several ways which proved equivalent by forming a tight cluster. Namely, the BoE was encoded as either a continuous variable (marked as breadth_continuous), discretized into three bins (breadth_discrete_3), discretized into 10 bins (breadth_discrete_10), or transformed and expressed as a square root (breadth_sqrt_1). [file 13059_2014_413_MOESM7_ESM.pdf]

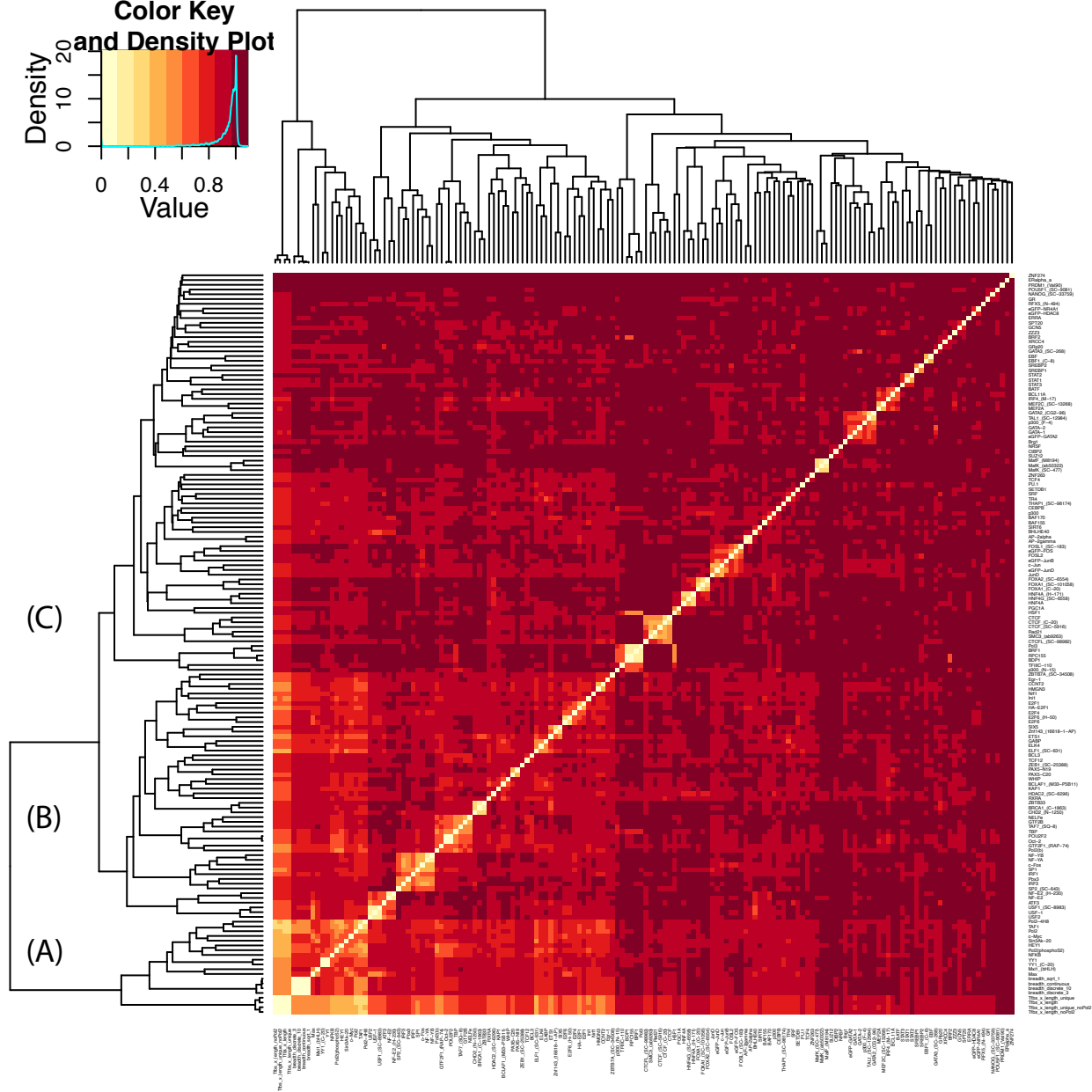

Supplement: Supplementary file 8 — The clustering of the BoE with the number of transcription factor binding sites in human tissues (Spearman’s rank correlation coefficient). Transcription factors clustering closest with the BoE were marked as A. Other transcription factors formed two clusters with low and high distance to the BoE (these clusters were marked as B and C, respectively). To test the robustness of this analysis, the number of transcription factors was measured in several different ways, which reassuringly clustered together and proved indistinguishable. The different measures were: sum of all sites (marked as Tfbs_x_length), sum of unique sites (Tfbs_x_length_unique), sum of all sites without RNA polymerase II (Tfbs_x_length_noPol2), and finally the sum of unique sites without the polymerase (Tfbs_x_length_unique_noPol2). The BoE was also transformed in several ways which proved equivalent by forming a tight cluster. Namely, the BoE was encoded as either a continuous variable (marked as breadth_continuous), discretized into three bins (breadth_discrete_3), discretized into 10 bins (breadth_discrete_10), or transformed and expressed as a square root (breadth_sqrt_1). [file 13059_2014_413_MOESM8_ESM.pdf]

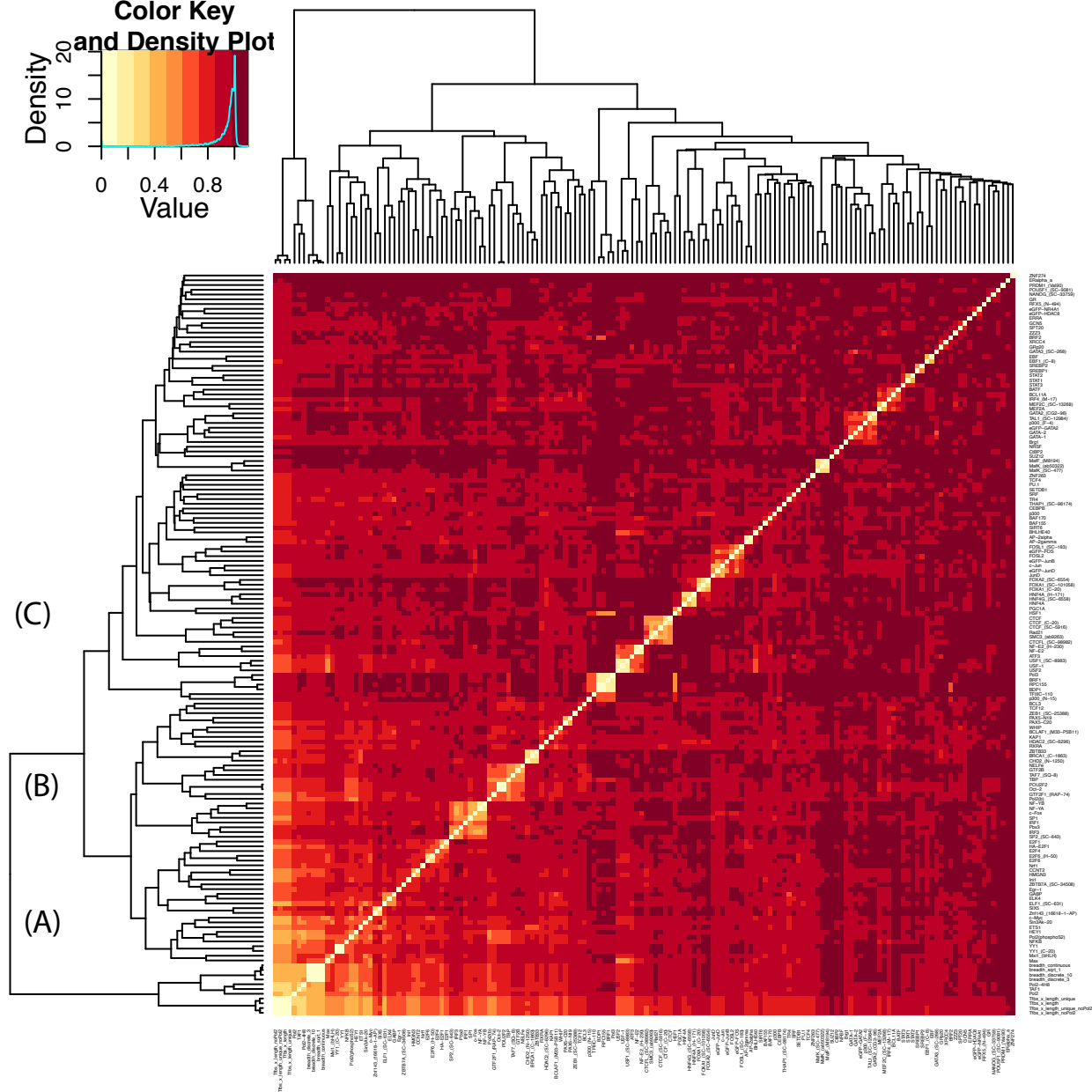

Supplement: Supplementary file 11 — The clustering of the BoE with the number of transcription factor binding sites in human cancer cell lines. In cancer cell lines, the BoE only clustered with RNA polymerase II and TFIID (this cluster was marked as A) suggesting that cancerous transformation disables most normal control switches for the BoE. Other transcription factors formed two clusters with low and high distance to the BoE (these clusters were marked as B and C, respectively). To test the robustness of this analysis, the number of transcription factors was measured in several different ways, which reassuringly clustered together and proved indistinguishable. The different measures were: sum of all sites (marked as Tfbs_x_length), sum of unique sites (Tfbs_x_length_unique), sum of all sites without RNA polymerase II (Tfbs_x_length_noPol2), and finally the sum of unique sites without the polymerase (Tfbs_x_length_unique_noPol2). The BoE was also transformed in several ways which proved equivalent by forming a tight cluster. Namely, the BoE was encoded as either a continuous variable (marked as breadth_continuous), discretized into three bins (breadth_discrete_3), discretized into 10 bins (breadth_discrete_10), or transformed and expressed as a square root (breadth_sqrt_1). [file 13059_2014_413_MOESM11_ESM.pdf]

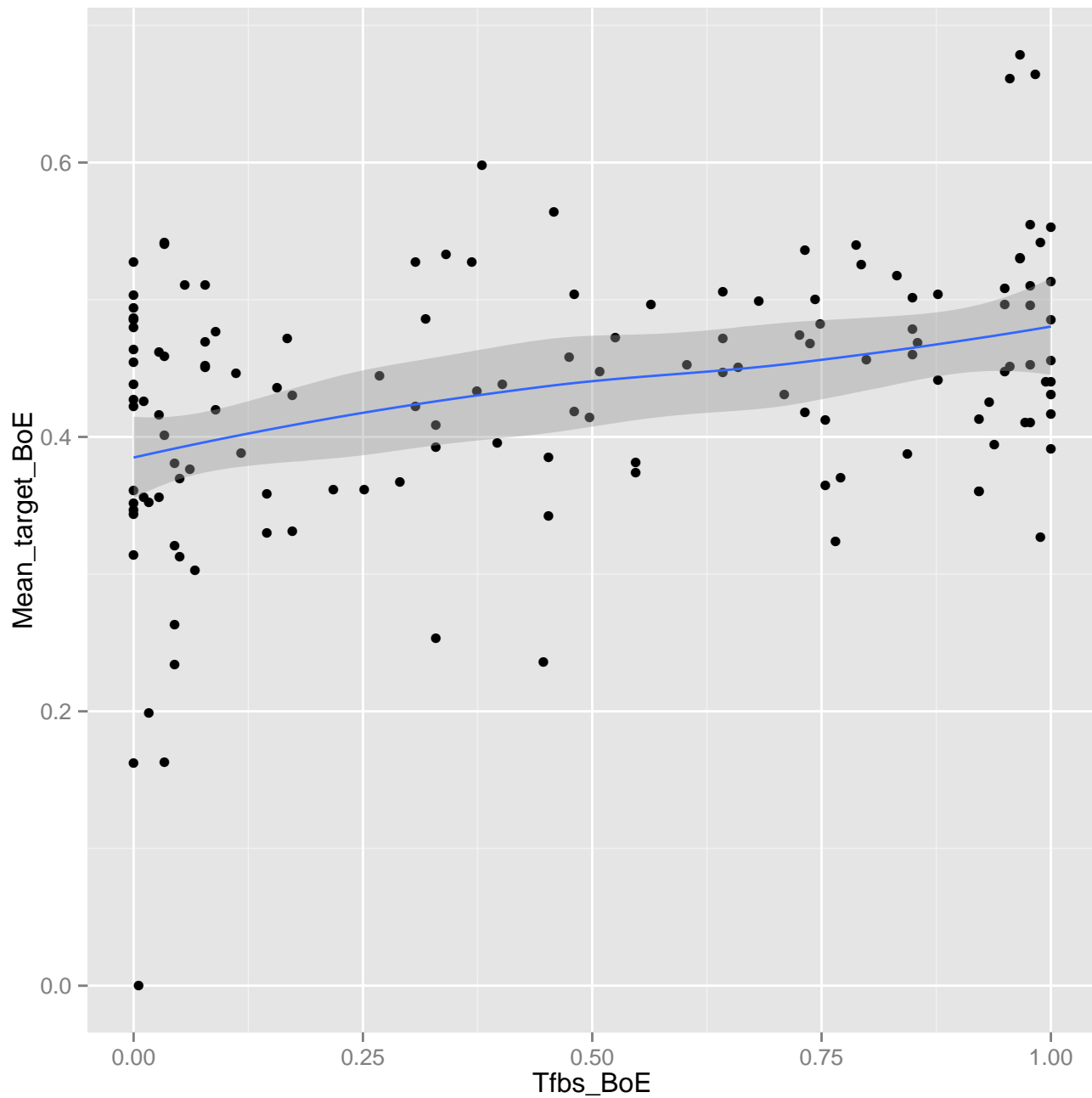

Supplement: Supplementary file 12 — There was a positive correlation between the BoE of transcription factors and the average BoE of their targets. The BoE of transcription factors in FANTOM5 tissues was plotted on the X-axis of the scatterplot (signified by Tfbs_BoE). The unweighted mean of the BoE of all target genes (that is, all genes that have a given Tfbs in their proximal promoter) was plotted on the Y-axis (signified by Mean_target_BoE). As both the independent and dependent variables were highly non-normally distributed, we used non-parametric correlation (Spearman’s rho = 0.3176). An alternative measure, a weighted mean, in which that data points were weighted according to the actual number of transcription factor binding sites was also considered and gave almost exactly the same correlation. The blue line is the fitted loess curve. [file 13059_2014_413_MOESM12_ESM.pdf]

**(a) ROC curve**

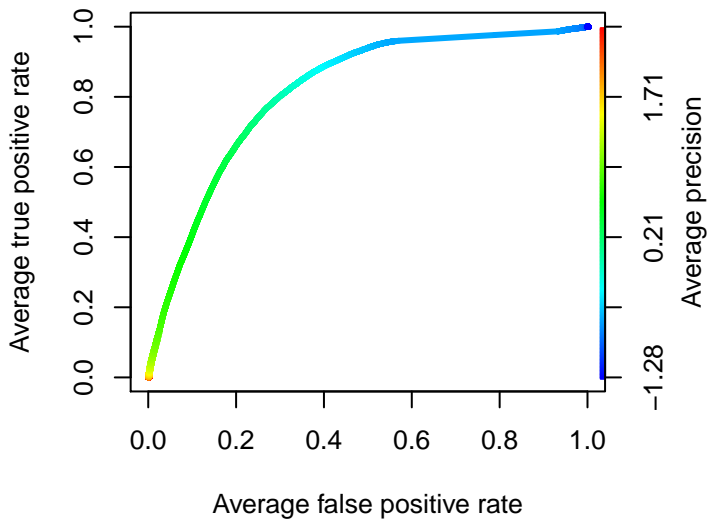

**(b) Precision/recall graph**

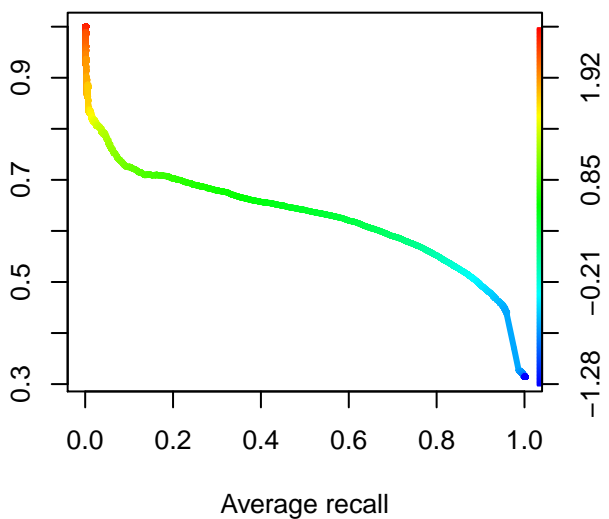

**(c) Sensitivity/specificity plot**

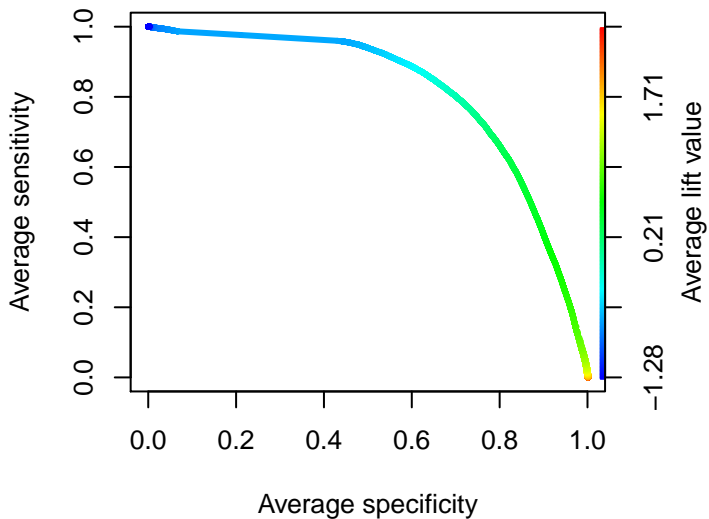

**(d) Lift chart**

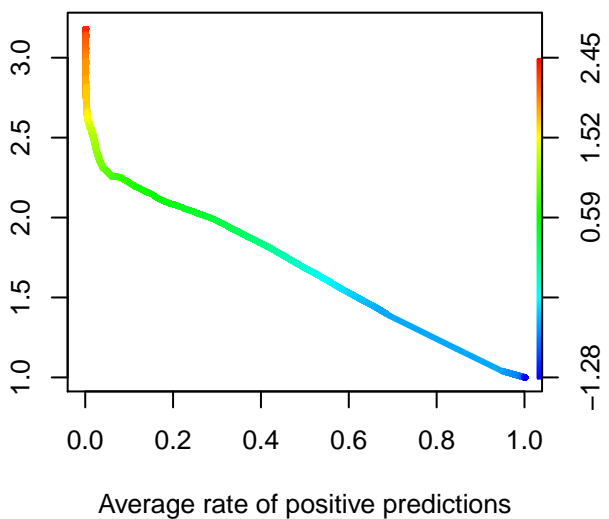

Supplement: Supplementary file 13 — Performance of SVM models as the predictor of tissue-specific (that is, narrow) expression. Tissue-specific expression was defined as BoE lower than 0.33 (that is, a gene that was expressed in less than one-third of tissues) and transcripts were categorized as either tissue-specific or not (that is, in a binary classification). This figure consists of four panels. The panels display standard predictor validation charts for the basic SVM model (SVM-Tfbs): (a) the ROC curve, (b) the precision/recall graph, (c) the sensitivity/specificity plot, and (d) the lift chart. The curves were averages from 10 different cross-validation runs. The area under the ROC curve equaled 0.816 (standard deviation equaled 0.002325). The parameterization of the curves was performed using the value of the linear SVM output and visualized by printing cutoff values at the corresponding curve positions (the curve was also colored according to the cutoff). The curves were plotted using R package ROCR. Essentially identical results were obtained a more complex SVM model with added data on GC content (SVM-Tfbs + GC), with AUC = 0.816. [file 13059_2014_413_MOESM13_ESM.pdf]
