# Supplementary material for: A simple metric of promoter architecture robustly predicts expression breadth of human genes suggesting that most transcription factors are positive regulators
Source: Genome Biol. 2014 Jul 31;15(7):413. doi: 10.1186/s13059-014-0413-3 (PMC4310617; doi:10.1186/s13059-014-0413-3)
Supplement: Supplementary file 5 — P values for pairwise BoE comparisons using Wilcoxon rank sum test for data in Table 9. NOTE: P value adjustment method: holm. [file 13059_2014_413_MOESM5_ESM.pdf]

TABLE S3. *P*-values for pairwise BoE comparisons using Wilcoxon rank sum test for data in Table 9.

	Primate	Mammalian	Vertebrate	Animal
Mammalian	<2e-16	-	-	-
Vertebrate	<2e-16	<2e-16	-	-
Animal	<2e-16	<2e-16	<2e-16	-
Eukaryotic	<2e-16	<2e-16	1e-12	<2e-16

NOTE: *P*-value adjustment method: *holm*.
