# Supplementary material for: A simple metric of promoter architecture robustly predicts expression breadth of human genes suggesting that most transcription factors are positive regulators
Source: Genome Biol. 2014 Jul 31;15(7):413. doi: 10.1186/s13059-014-0413-3 (PMC4310617; doi:10.1186/s13059-014-0413-3)
Supplement: Supplementary file 6 — P values for pairwise TfbsNo. comparisons using Wilcoxon rank sum test for data in Table 10. NOTE: P value adjustment method: holm. [file 13059_2014_413_MOESM6_ESM.pdf]

TABLE S4. *P*-values for pairwise TfbsNo. comparisons using Wilcoxon rank sum test for data in Table 10.

	Primate	Mammalian	Vertebrate	Animal
Mammalian	1.1e-15	-	-	-
Vertebrate	< 2e-16	< 2e-16	-	-
Animal	< 2e-16	< 2e-16	8.5e-10	-
Eukaryotic	< 2e-16	< 2e-16	< 2e-16	< 2e-16

NOTE: *P*-value adjustment method: *holm*.
