# Supplementary material for: A simple metric of promoter architecture robustly predicts expression breadth of human genes suggesting that most transcription factors are positive regulators
Source: Genome Biol. 2014 Jul 31;15(7):413. doi: 10.1186/s13059-014-0413-3 (PMC4310617; doi:10.1186/s13059-014-0413-3)
Supplement: Supplementary file 10 — The clustering of the BoE with the number of transcription factor binding sites in human primary cells. In human primary cells, the BoE clustered with the polymerase, TFIID, NFKB, HEY1, Sin3A, and c–Myc (this cluster was marked as A). Other transcription factors formed two clusters with low and high distance to the BoE (these clusters were marked as B and C, respectively). To test the robustness of this analysis, the number of transcription factors was measured in several different ways, which reassuringly clustered together and proved indistinguishable. The different measures were: sum of all sites (marked as Tfbs_x_length), sum of unique sites (Tfbs_x_length_unique), sum of all sites without RNA polymerase II (Tfbs_x_length_noPol2), and finally the sum of unique sites without the polymerase (Tfbs_x_length_unique_noPol2). The BoE was also transformed in several ways which proved equivalent by forming a tight cluster. Namely, the BoE was encoded as either a continuous variable (marked as breadth_continuous), discretized into three bins (breadth_discrete_3), discretized into 10 bins (breadth_discrete_10), or transformed and expressed as a square root (breadth_sqrt_1). [file 13059_2014_413_MOESM10_ESM.pdf]

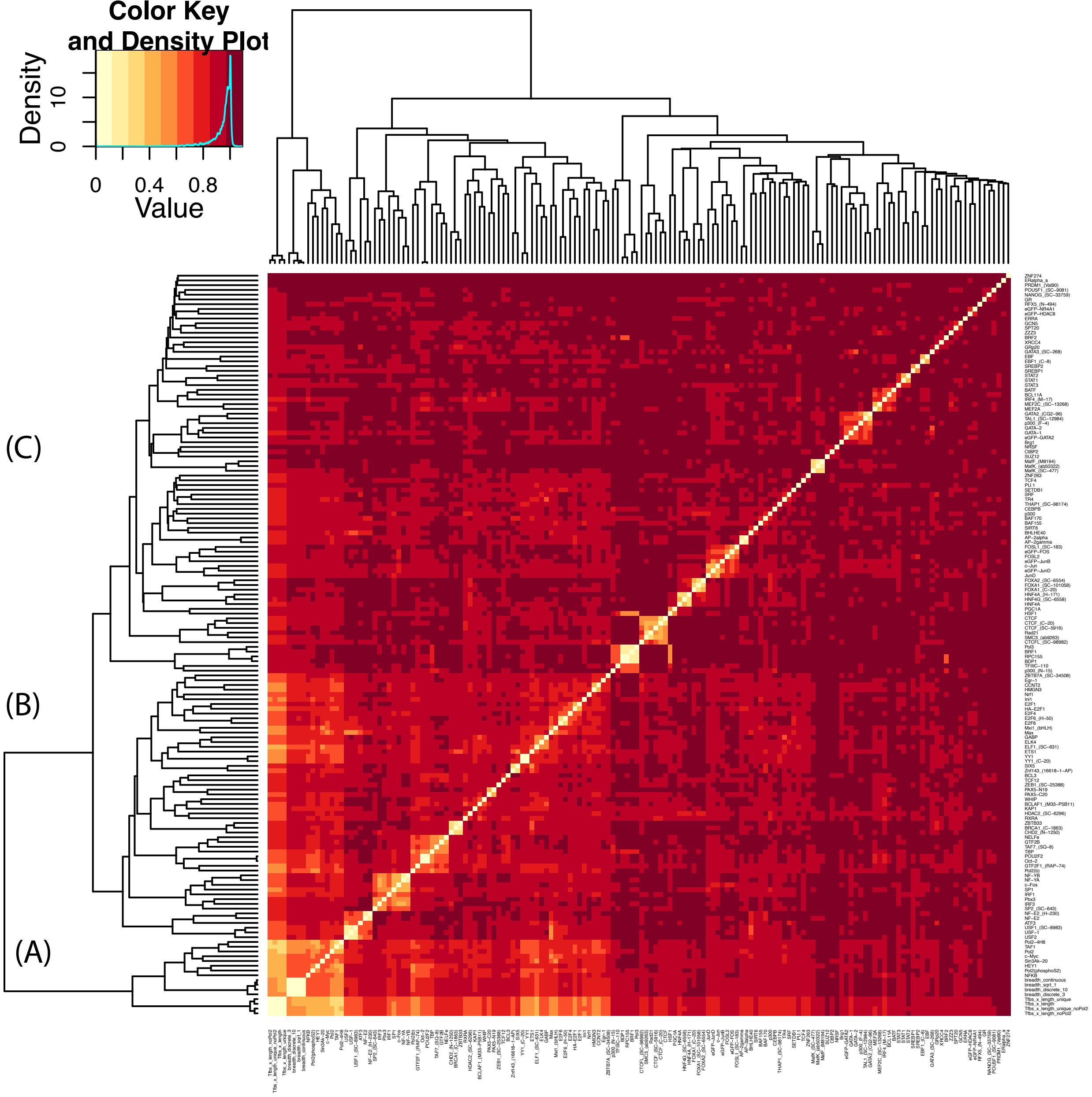

Color Key
and Density Plot
Density
0
10
0
0.4
0.8
Value
(C)
(B)
(A)
